# Embedding Chemistry and Pharmacy Into Sustainability

**DOI:** 10.1002/anie.3329101

**Published:** 2026-05-11

**Authors:** Klaus Kümmerer

**Affiliations:** ^1^ Institute for Sustainable Chemistry Leuphana University Lüneburg Germany

## Abstract

Chemistry and pharmacy provide products and processes that are essential to our high standard of living. Chemistry is the central science when it comes to the transformation of substances, in pharmacy too. Against the backdrop of sustainability, both chemistry and pharmacy face similar challenges. They have taken up aspects of environmental friendliness, circular economy, and sustainability. However, the term “green” is often used synonymously with “sustainable” or even combined as “green and sustainable,” even though sustainability can not be considered synonymous with “green.” Sustainable chemistry and pharmacy recognize that green and circular chemistry and pharmacy are important tools. They, however, go far beyond. This article provides an integrated overview of green, circular, and sustainable chemistry and pharmacy to clarify their respective areas of application and the respective interplay between “green” and “circular” and “sustainable” so that both, chemistry and pharmacy can fulfill their promise of delivering sustainable contributions to sustainability in a sustainable manner. This article illustrates that it is not sufficient simply to place sustainability alongside chemistry or pharmacy—“for” or “and” sustainability—but that chemistry and pharmacy must be fully embedded into sustainability.

## Graphical abstract

Chemistry and pharmacy provide products and processes that are indispensable for our high living standard. To understand their relationship with sustainability is important to allow them to contribute to sustainability in a sustainable manner. An integrated overview of green, circular, and sustainable chemistry and pharmacy is given and how they have t embedded into sustainability.

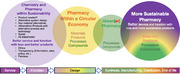

## 1 Introduction

Chemistry is the science of the transformation of substances. As such, it is also an important pil of pharmacy. Chemistry and pharmacy are therefore indispensable pillars of our high standard of living and our health [1, 2, 3]. More than 95% of today's products are based on at least one chemical reaction [4]. In recent decades, demand for products has risen enormously, accompanied by a sharp increase in the consumption of natural resources such as minerals, metals, biological resources, and fossil carbon for the synthesis and manufacture of products [5, 6, 7]. All non‐radioactive elements of the periodic table and even some of the radioactive elements are used today [8]. Global turnover from chemicals is expected to almost double between 2020 and 2030, reaching €6.6 trillion [9]. Sales of pharmaceuticals are also rising rapidly and, according to Statista, are expected to grow from 1.7 trillion US dollars in 2024 to around 2.4 trillion US dollars in 2029. This will be reflected in a further increase in resource demand, waste, and impacts on health and the environment.

More than 300,000 chemicals are marketed worldwide [10]. Within the group of per‐ and polyfluoroalkyl substances (PFAS), the so‐called “forever chemicals,” around 10 000 different compounds are in use, and many more are known [11, 12]. New materials have been introduced (e.g., graphene, nanoparticles, metal‐organic frameworks (MOFs), or materials for electronics). Polymers, fibers, additives, and excipients have become increasingly complex and diverse at the atomic to the molecular level. Plastics, for example, are rarely simple polymers such as polyethylene. Elements such as oxygen, nitrogen, or sulfur are incorporated into the hydrocarbon backbone in both block and non‐block configurations, and various functional groups are present regularly and irregularly at the molecular level. One‐dimensional chains form two‐ or three‐dimensional molecular networks. In addition, more than 10 000 additives, that is, various chemicals, are used to modify the properties of the polymers [13, 14]. Such polymers are combined, for example, in high‐performance materials and products consisting of a mixture of several polymers and additives, rather than a single, pure polymer. Around 3000 active pharmaceutical ingredients are currently in use, in addition to numerous excipients and adjuvants that form part of medicines. The individual constituents, that is, active or main components of pesticides, disinfectants, dyes, polymers, fibers, additives, and excipients, have become increasingly complex and diverse at the atomic and molecular levels. In the related products such as alloys, pesticides, washing agents, paints, plastics, medicines, electronics, textiles, paper, tires, cars, furniture, and buildings, chemicals and materials are increasingly mixed. In other words, they have become increasingly complex and diverse at the atomic to the molecular to the material to the building block to the product level. A car tire, for example, contains 40%–50% “polymeric material,” 30%–35% fillers, 15% plasticizers, 2%–5% vulcanizing agents and 5%–10% other additives [15]. Mobile phones and other electronic devices contain up to 60 chemical elements, that is, metals, metalloids, and non‐metals, each in different chemical compounds at varying oxidation states, together with polymers, flame retardants, ceramics, and other chemicals and materials. Textiles may contain various dyes, fibers, polymers, and other chemicals. In most cases, the exact composition and the number of added substances are unknown to recycling companies. Each of these products also contains residues of mostly unknown non‐intentionally added substances (NIAS), which arise during raw material processing, synthesis, and manufacture, as well as residues resulting from use and aging.

The undesirable side effects of the chemical industry became apparent at an early stage: environmental pollution in the form of emissions from chimneys, odors and colored river water, but also in the form of health risks and resource consumption. Chemical waste, for example, from synthesis, was for a long time simply disposed of or discharged into rivers, thereby poisoning people and organisms in the environment. A notorious example is the Cuyahoga River in Cleveland (Ohio, USA) [16]. It was so heavily polluted with chemicals that it caught fire in 1969 and burned for about 20 min. This resulted, among other things, in damage to two railway lines and the contamination of the local air and soil with pollutants. Another notorious case is the Love Canal in Buffalo (New York, USA), which came to light in the late 1970s. For decades, this site was used as a chemical waste tip before being built over. The local population's exposure to toxic chemicals caused numerous health problems, including birth defects, miscarriages, epilepsy, and leukemia [17]. Many other examples of “unregulated” or inadequately secured hazardous waste disposal sites “discovered” in other countries during the 1980s could be mentioned here (e.g., Lekkerkek in the Netherlands, Vac in Hungary, and Georgswerder or Malsch in Germany). This illustrates the common practice at the time regarding the handling of chemical waste. In some countries, such hazardous practices of landfilling chemical waste or incinerating it in unsuitable facilities are still in use, leading, among other things, to the emission of carcinogenic chemicals such as polycyclic aromatic hydrocarbons (PAHs) and polychlorinated dioxins and furans (see below).

Some of the most toxic synthetic chemicals have been identified as unintended by‐products of the synthesis of chlorinated organic chemicals, such as pesticides. For example, 2,4,5‐trichlorophenol is an intermediate in the production of hexachlorophene, which was once widely used as a disinfectant and in the manufacture of herbicides, as well as 2,4,5‐trichlorophenoxyacetic acid. This was used as an herbicide and defoliant and was also employed by the US military as a chemical warfare agent (e.g., “Agent Orange”) to defoliate trees during the Vietnam War. A by‐product of the 2,4,5‐trichlorophenol synthesis is 2,3,7,8‐tetrachlorodibenzodioxin (“Seveso Dioxin”, TCCD), one of the most toxic chemicals known to humans. Agent Orange contained Seveso Dioxin as an undesirable but known by‐product of the synthesis (“contamination”). The consequences of the poisoning of the local population and US Army soldiers are still visible today in the second generation. In the Seveso disaster—named after the Italian town where the accident occurred—TCCD was released due to a reaction that got out of control. Other well‐known incidents that could be cited as examples include the accidents at Schweizerhalle (Switzerland) and Bhopal (India), both of which occurred in the 1980s. Seveso Dioxin and other related chlorinated dioxins and chlorinated dibenzofurans are also produced during the incineration of organic waste in the presence of chlorine. Another by‐product of the combustion of organic materials is PAHs, which are very toxic and, as for some compounds of the group, highly carcinogenic.

As early as 1962, Rachel Carson highlighted the undesirable effects of certain products, such as pesticides and medicines, on the local environment [18]. By the 1960s and 1970s, environmental chemistry had already developed to the point where it incorporated systemic approaches and was not merely concerned with identifying chemical pollution of the environment, but was already looking further ahead to consider how environmental pollution could be prevented from the outset and what new alternative energy sources might be necessary, or the depletion of mineral resources and its consequences [19]. As early as 1973, the depletion of stratospheric ozone by synthetic chlorofluorocarbons (CFCs) was recognized as a global chemical impact [20]. Despite this knowledge, however, production rose sharply after 1973 until the Montreal Protocol came into force in 1987 [21]. This 14‐year delay between the availability of the knowledge and the taking of action is responsible for more than 90% of the pollution caused by CFCs, the effects of which will persist until the end of this century.

Alongside numerous other substances, organic chemicals containing fluorine, chlorine, or bromine—including many pesticides, some pharmaceuticals, PFAS, and flame retardants—persist in the environment for long periods [22]. Even decades later, many of these chemicals are still present in the environment or in products and are often euphemistically referred to as “legacy chemicals” or “contaminated sites.” The most recent example is PFAS. Although they entered the market decades ago and have long been known for their undesirable properties, they have only recently received wider attention [11, 23, 24, 25, 26]. Raw material extraction and waste treatment, including recycling, still contribute significantly to environmental pollution and high water consumption. Chemical pollution of the environment and food contamination have also reached an all‐time high in terms of chemical diversity.

In other words, the downside of the more than 200‐year success story of (industrial) chemistry is that, alongside the desired products, their unwanted by‐products can also be found everywhere around us—that is, in the environment—and within us, some of them having been present for several decades [27, 28, 29, 30, 31, 32, 33, 34]. For the vast majority of chemicals and pharmaceuticals, as well as for numerous unwanted by‐products arising during synthesis, processing, use, and in the environment, data on toxicity, environmental fate, and effects are often very limited. Despite our growing knowledge, we know less than ever about the fate and effects of chemicals and pharmaceuticals in the environment, in food, and in organisms, compared to what we ought to know. The necessary increase in knowledge cannot keep pace with the enormous growth in substances, materials, products, and applications.

The use of certain environmentally persistent organic pollutants (POPs) was banned under the Stockholm Convention, which came into force in 2004 [22]. The list has been growing steadily ever since. It began with the so‐called “Dirty Dozen,” including polychlorinated biphenyls (PCBs), highly chlorinated pesticides, and Seveso Dioxin, which, due to their long half‐lives, are still present in the environment, in food, and in the human body. This was followed by additional groups of chemicals, such as brominated flame retardants and, more recently, some PFAS. All preventive measures were taken only after their environmental persistence and their toxic properties had already been demonstrated years or even decades earlier.

Resources for remediation measures are generally insufficient or even non‐existent, leading to additional, often hidden costs and a continuing burden on people and the environment. The annual health‐related costs for PFAS alone have been estimated at between €52 and 84 billion in the European Economic Area [23] and at US$60 billion in the USA [24]. The costs for environmental monitoring alone are estimated at a total of €170 billion, yet monitoring alone will not change anything at all. The US Water Works Association estimates the cost of removing PFAS from drinking water at US$370 billion [25]. While the average market price for PFAS in 2022 was 19€/kg, the actual price is dramatically higher, reaching 18 700€/kg if social costs such as those mentioned above are taken into account [25]. Although all these cost estimates are subject to a degree of uncertainty and depend on certain assumptions, for example, regarding PFAS usage rates and concentrations in soil or water, the figures once again highlight the high follow‐on costs of past inaction. From a sustainability perspective, these costs, as well as the health and environmental impacts of the chemicals, should be prevented in advance, as should the resulting unquantifiable harm to people and nature. These follow‐on costs must until now generally be borne by society, not by companies that made the money. The money saved through precautionary measures and behavior could be used for other purposes, including combating poverty or improving education and training, including in the fields of sustainability and chemistry.

**FIGURE 1:**
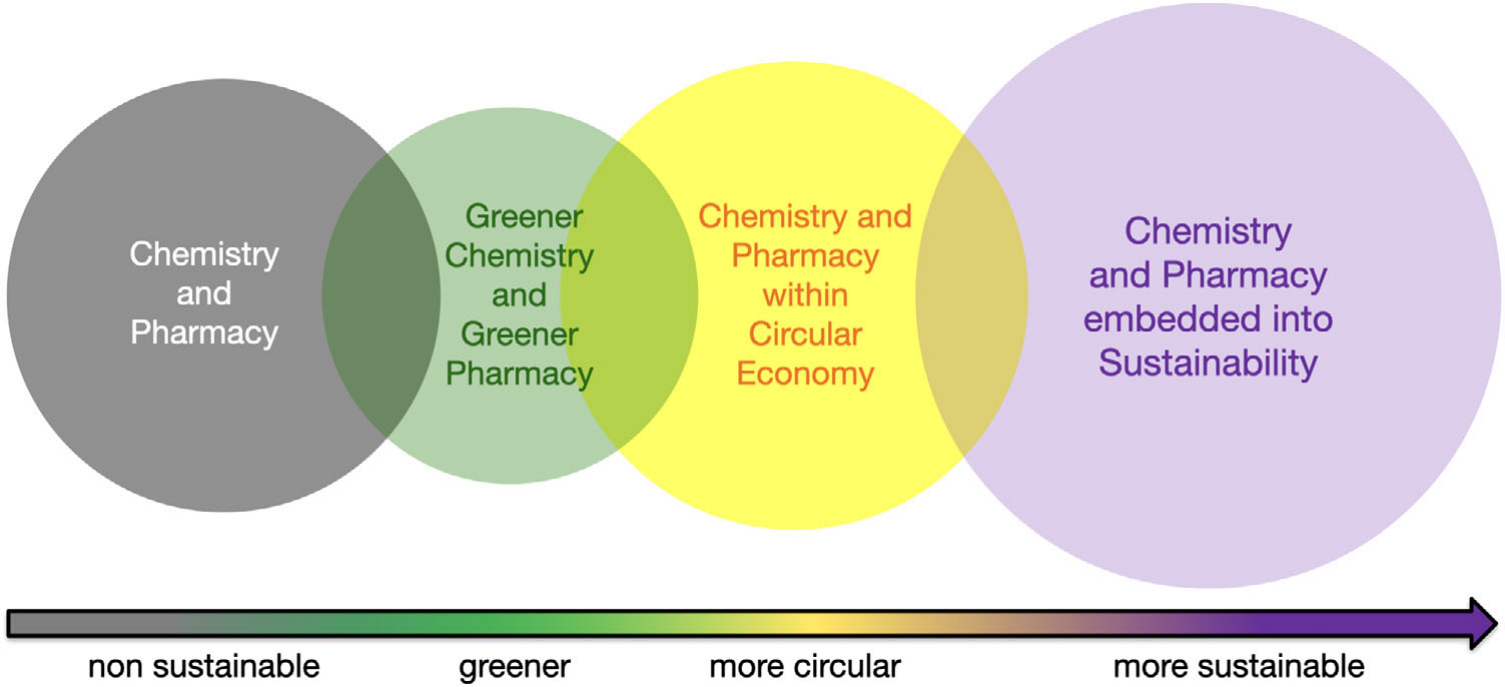
Historical development of chemistry and pharmacy against the backdrop of sustainability.

The concept of the circular economy was developed in response to the need to prevent environmental pollution and address resource scarcity through the recycling of waste, including strategic raw materials such as rare earth elements (REEs), graphite, and other critical minerals. Both green chemistry and circular chemistry address environmental pollution, hazards, and resource issues associated with the manufacture and use of chemicals and pharmaceuticals. Sustainable chemistry and sustainable pharmacy recognize these as important tools for more sustainable chemistry and pharmacy, while at the same time highlighting their inherent limitations. Sustainable pharmacy differs from sustainable chemistry in certain respects, for example, in some ethical ones [35, 36]. Green approaches based on the use of biologically sourced remedies have long been part of pharmacy (e.g., plant‐based active ingredients, pharmaceutical biology), but have not progressed much further. However, as regards resource use, synthesis and end‐of‐life issues relating to their products, as well as their application and their connection to sustainability, chemistry and pharmacy have much in common. The historical development is summarized in Figure 1.

The terms “green chemistry” and “green pharmacy” are often used synonymously with sustainable chemistry and sustainable pharmacy, respectively. The same holds for their products. The circular economy is often viewed as a source of infinite resources, as supposedly everything can be recycled. However, this reflects the traditional thinking of the linear economy. Not everything that is greener or more suitable for a circular economy is necessarily more sustainable. In other words, the terms “green,” “circular,” and “sustainable” are often used without a clear understanding of what they mean (see also Chapter 6). There is a lack of clear understanding of the respective opportunities, interrelationships, limitations, pitfalls, and areas of application. Yet this knowledge is the indispensable foundation for avoiding misunderstandings that lead to processes and products that are not green, not circular, or not sustainable.

This article, therefore, aims to provide a much‐needed better understanding of the opportunities and limitations of these concepts—that is, their respective areas of application and how they are interlinked and can support one another—so that the chemical and pharmaceutical industries can contribute to a more sustainable future in a more sustainable way and avoid rebound effects, greenwashing, and sustainability‐washing (i.e., the promotion of supposedly greener solutions and products that are neither greener nor more sustainable).

## 2 Chemistry and Pharmacy: Science and Industrial Application

**FIGURE 2:**
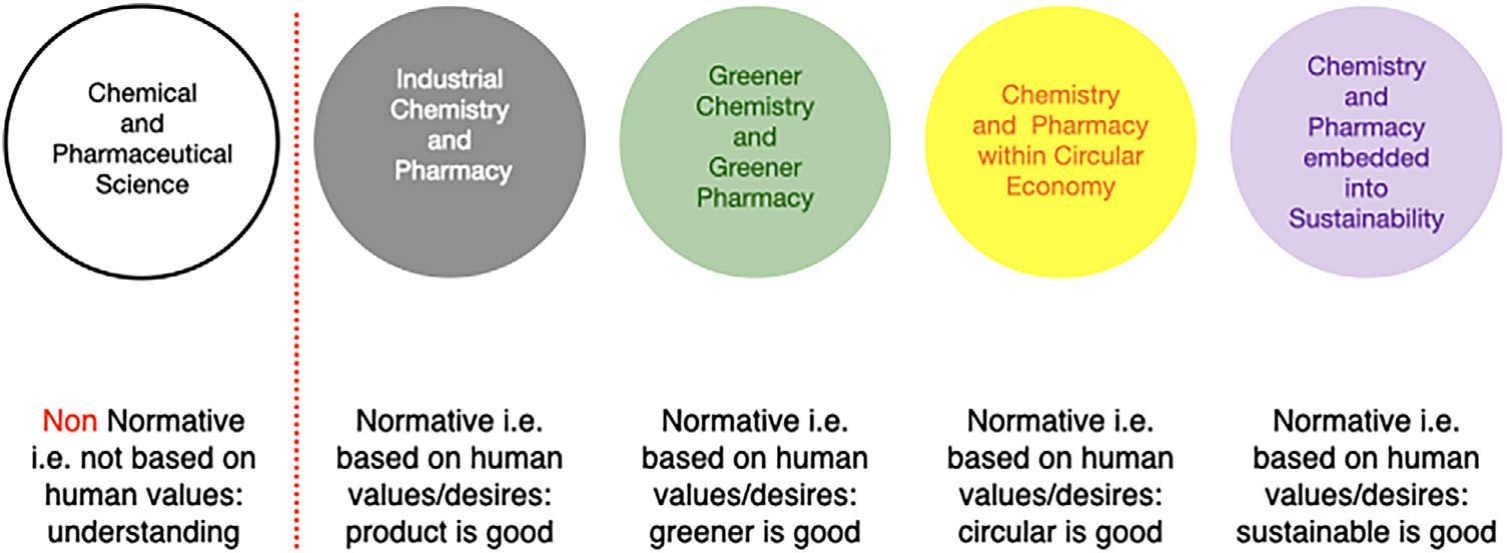
Non‐normative and normative chemistry and pharmacy.

**FIGURE 3:**
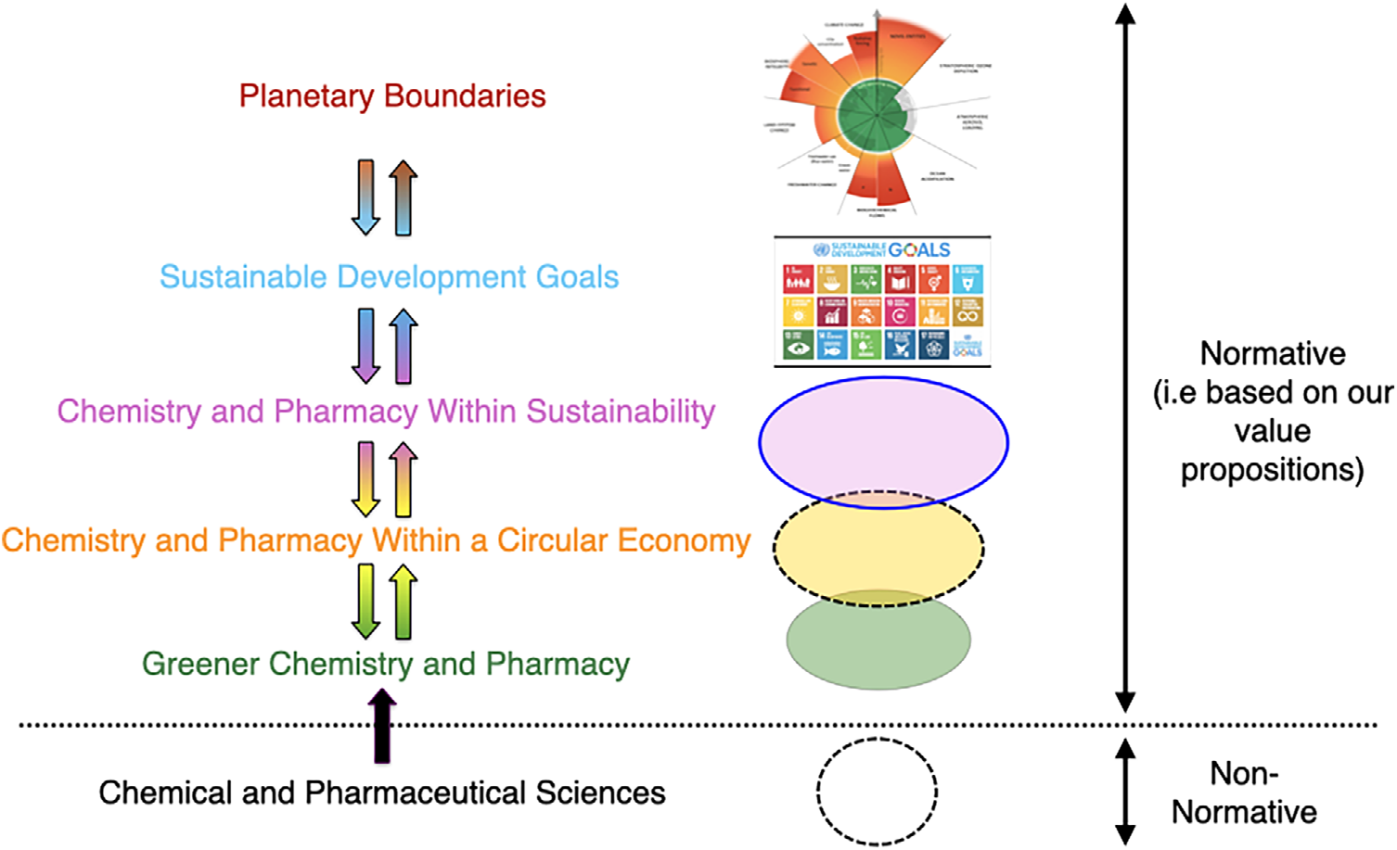
The interrelationships between chemistry and pharmacy as sciences, green, circular, and sustainable approaches, the Sustainable Development Goals, and Planetary Boundaries (https://www.stockholmresilience.org/research/planetary‐boundaries.html; figure “Planetary Boundaries” licensed under CC BY‐NC‐ND 3.0. *Source*: Azote for the Stockholm Resilience Centre, Stockholm University; based on Sakschewski and Caesar et al. 2025). SDGs: https://sdgs.un.org/goals. SDGs image overlay: https://www.umweltbundesamt.de/sites/default/files/medien/377/bilder/sdgs_poster_new1.png.

In an industrial context, we synthesize chemicals and materials with a specific benefit, function, or business opportunity in mind. Unlike the fundamentals of chemistry and pharmacy as sciences, we can alter our individual and societal values and expectations regarding industrial chemistry and pharmacy, as these are not determined by nature (Figure 2). For example, based on the costs of separating and extracting components related to the hazards posed by the ingredients, we can decide whether to classify a particular type of waste as “good” (e.g., a resource to earn money through “upcycling,” see Chapter 4.4.3) or as “bad” (hazardous, expensive to dispose of). Or we may decide that renewable resources are “better” than non‐renewable ones, or that the introduction of chemicals into water bodies is undesirable (leading to pollution) or desirable (e.g., algaecides). The same applies to the so‐called planetary boundaries (PBs, see Chapter 5), including those relating to chemicals. They are not defined by nature, but by our human values, that is, the desire to ensure a habitable planet for us. The same applies to the Sustainable Development Goals (SDGs, see Chapter 5). In other words: when it comes to the industrial application, i.e., the synthesis of compounds and materials as well as the manufacture of products etc, chemistry and pharmacy become normative, that is, they are determined by human values such as environmental friendliness, the circular economy, sustainability, business opportunities, the SDGs, or the PBs (Figure 3). For nature, none of these terms and categorizations play a role, and we cannot derive them from nature. That would be to succumb to the naturalistic fallacy. The properties and behavior of atoms, molecules, materials, and products are inherent in their composition. We cannot alter them. In contrast, values and desires can change, as they are not given by nature.

## 3 Pollution Prevention and Green Chemistry

### 3.1 Greener Chemistry From the 1970s to the Early 1990s

The synthesis‐oriented concept of pollution prevention was developed and commercialized by industry in many countries as early as the 1970s and 1980s. This development arose in response to environmental pollution caused by chemical waste and the associated regulations and costs [37, 38, 39, 40, 41, 42, 43, 44, 45]. It also aimed to prevent the incomplete degradation of chemicals in the environment [46]. This was triggered by a growing understanding of the presence and potential effects of chemicals in the environment, in food, and on human health. Insights gained thanks to the new disciplines of environmental chemistry and ecotoxicology, which emerged in the 1960s and 1970s. Both disciplines already applied systems thinking; for example, environmental chemistry was fundamentally linked to the prevention of environmental pollution [19]. Further drivers of this development were impending legislation and rising costs associated with chemical waste and liability claims. For example, under the US Superfund, polluters were required to pay for decontamination, regardless of the history of the pollution and the regulations in force at the time of the incident. Several increasingly environmentally friendly chemical processes were developed and introduced into synthesis and manufacturing, that is, commercialized at that time [37, 38, 39, 40, 41, 42]. This was supported by contributions from research [47, 48, 49, 50].

The UN report published in 1987, entitled “Our Common Future” (the “Brundtland Report”), addressed chemical pollution and its impacts and noted already then that around 80 000 different chemicals had been placed on the market during the 1980s. The report emphasized that a life‐cycle perspective was necessary in chemicals management and that measures needed to be taken to reduce risks associated with chemicals and chemical waste, with particular mention of “non‐chemical methods of pest control” [51]. In 1989, the Toxic Use Reduction Act came into force in the US state of Massachusetts, and the Toxic Use Reduction Institute was established at the same time. A year later, the Pollution Prevention Act came into force in the USA, which explicitly named “pollution prevention as an environmental goal for the 1990s” in order to (i) reduce the quantity of hazardous substances, pollutants, or contaminants entering the waste stream or otherwise being released into the environment prior to recycling, treatment or disposal, and (ii) to reduce the risks to public health and the environment associated with the release of substances, pollutants, or contaminants [52, 53]. In the early 1990s, many different organizations, institutions, and individuals devoted themselves to the prevention of waste and environmental pollution caused by chemical synthesis. In 1992, the Rio Declaration—a collection of 27 principles for steering sustainable development, which was incorporated into UN Agenda 21—highlighted the importance of intensified research into the development of safe substitutes for chemicals with long lifecycles [53]. In summary, it can be said that the understanding of pollution prevention and risk reduction in the 1980s and early 1990s was often much more comprehensive than what is today referred to as “greener” synthesis and the now widespread technology‐oriented understanding of green chemistry and green technology.

In the 1990s, several organizations, including the US Environmental Protection Agency (EPA), the OECD, the EU, numerous national chemical societies (such as the German Chemical Society), industry associations, and many individuals, proposed various terms, approaches, and tools for preventing environmental pollution caused by chemicals [47, 53, 54, 55, 56, 57, 58]. “Multiple green chemistries struggled for recognition, both within the EPA and beyond, with a tremendous amount of theoretical reflection on how to combine sustainability with chemistry conducted in Europe. In Italy, France, and Germany, many scholars anticipated what would become green chemistry in parallel to their American colleagues … These scientists were not merely imperfect forerunners to a more mature EPA‐based concept but rather developed full‐fledged alternatives, many of which have a lot to offer to modern practitioners of green chemistry” [58]. The term “green chemistry” was not invented by the US EPA but was promoted by the agency from the early 1990s on “… placing the birth of green chemistry at the EPA in 1991, a year from which there is not a single publication on the topic, appears to be problematic” [58]. The term “green chemistry” was probably used for the first time in 1987 by two Italians, Pasquon and Zanderighi, who published a textbook entitled *La Chimica Verde* (Green Chemistry) [59]. The first known industrial program on “green chemistry” to be explicitly named as such was funded in 1989 by the Italian chemical company Ferruzzi and attracted international attention [60].

In 1996, Council Directive 96/61/EC of the European Community (EC) came into force as an important legal instrument dealing with the “integrated prevention and control of pollution” in industry—not just the chemical industry [61]. Annex III of the Directive lists groups of chemicals that should be avoided. The application of best available techniques, efficient use of energy and resources, the use of less hazardous compounds, and the prevention of accidents and their consequences are set out in Annex IV (see Table 1). It is particularly noteworthy that this regulation—the first of its kind, that is, a statutory provision—also emphasizes the need to avoid global and long‐term negative impacts on the environment (such as the protection of the ozone layer). Four of the principles listed in Annex IV bear a strong resemblance to the 12 principles of green chemistry (Table 1) [62]. This is understandable, as they are inherent to chemistry and industrial manufacturing, which are based on the same historical roots and experiences. In contrast to the approaches addressed by the 12 principles of green chemistry, the Directive aimed from the outset at synthesis and production on an industrial scale, including the associated industrial sectors, not just the chemical industry. Both the 12 principles in Annex IV of the directive and those of green chemistry, while differing in origin, deal with safer synthesis using fewer hazardous chemicals, less energy, and the generation of less waste to prevent environmental pollution and reduce the environmental impact. Both the EC Directive and the 12 principles of green chemistry are important approaches that can help make the chemical and pharmaceutical industries greener regarding the synthesis of individual chemicals. However, in contrast to the 12 principles of green chemistry, the EC Directive includes not just chemicals but allied industries and downstream users of their products. On top of this, it legally mandates the integration of measures to prevent environmental pollution into the entire chemical and other industrial production processes from a more holistic perspective, whereas the 12 principles of green chemistry are merely voluntary in nature, less comprehensive, and were only published 2 years later. Furthermore, the concept of green chemistry, as advocated by the US EPA and by Anastas and Warner, has been criticized for failing to address the broader challenges of the techno‐economic system responsible for environmental failure [43].

**TABLE 1:** Approaches for a more environmentally friendly and circular chemistry (abridged).

	Twelve principles of Annex IV to Council Directive 96/61/EC [61] Published in 1996 Precaution and prevention	Twelve principles of Green Chemistry [62] Published in 1998 Prevention of environmental pollution	Twelve principles of Circular Chemistry [109] Published 2019 Recycling
1	Low‐waste technology	Waste prevention	Waste collection and recovery
2	Less hazardous substances	Use of all materials used in the product	Maximized atom circulation
3	Recovery and recycling of substances and waste generated and used in the process	Lower toxicity of products	Optimized resource efficiency
4	Advanced processes, plants, or methods that have been successfully tested on an industrial scale	Fewer auxiliary materials	Energy efficiency
5	Technological advances and changes in scientific knowledge and understanding	Safer solvents and auxiliary materials	Improved process efficiency
6	Nature, impact, and extent of the emissions in question	Minimized energy requirements	No off‐site toxicity
7	Commissioning dates for new or existing plants	Renewable raw materials	Targeted, optimal design
8	Time required to implement the best available technology	No unnecessary derivatization	Sustainability assessment
9	Consumption and type of raw materials used in the process, as well as their energy efficiency	(Selective) catalysis	Circular economy model (“recycling ladder”)
10	Avoidance or minimization of the overall environmental impact and risks posed by emissions	Design for degradation	Service instead of product
11	Accident prevention and minimization of environmental consequences	Real‐time analysis for hazard prevention	Number of lock‐ins
12	Information published by the Commission pursuant to Article 16(2) or by international organizations	Substances to minimize accidents during synthesis	Coherent policy framework

In fact, none of the 12 principles, including the term “green chemistry,” were new at the time of their publication, as explained above, even though different terminology was used in some publications [37, 38, 39, 40, 41, 42]. Since the inception of pollution prevention, there have been many roots and developments, extensive research, and numerous contributors and supporters of what is now referred to as green chemistry. In fact, there were often even more and far‐reaching ideas, but these did not find their way into the 12 principles of green chemistry as published by Anastas and Warner. Collins, for example, published a more comprehensive and interdisciplinary understanding of green chemistry in 1997, which, whilst incorporating the 12 principles, did not designate them as principles [66]. In other words, “green chemistry” and the 12 principles as published by Anastas and Warner were not new concepts from the outset but rather a narrowly defined s; nor is there a “father” of green chemistry [63, 64, 65]. Instead, there were many individuals and institutions to whom the application of various measures to prevent environmental pollution can be attributed, some of which went far beyond what was later termed green chemistry. In their 1998 publication, Anastas and Warner summarized, from a narrower perspective, much of what industry, academia, government bodies, international organizations and others had long since published and applied in practice, either individually or collectively, including many examples of pollution prevention technologies [37, 38, 39, 40, 41, 42, 43, 44, 45, 46, 47, 48, 49, 50, 51, 54, 59, 60].

### 3.2 Sustainable Chemistry Before “green Chemistry”—Roots in the 1970s to the 1990s

A hint of sustainability was already evident in the publications and measures of the 1970s and 1980s, becoming clearly visible in the late 1980s and early 1990s [18, 19, 39, 45]. The Brundtland Report (1987) and the Rio Declaration (1992), for example, went even further than simply calling for fewer hazardous chemicals and less chemical waste by also addressing what we would today describe as alternative, non‐chemical business models. Pasquon and Zanderighi had already pointed out the unsustainable nature of fossil fuels, as well as the environmental impacts and destruction caused by their exploitation, which represents a far more comprehensive perspective than merely calling for renewable energy because fossil resources are finite [59]. The two scientists emphasized the need for plant‐based chemistry and argued that this was environmentally friendly due to the biodegradability and low toxicity of such products. However, they were also aware that not all plant‐based products are necessarily less toxic and that the cultivation of these resources requires additional resources, which negatively impact biodiversity, among other effects. In 1989, von Gleich introduced “*Sanfte Chemie*” (Gentle Chemistry) as a more comprehensive approach than merely avoiding environmental pollution or approaches that focus solely on more environmentally friendly chemistry [67]. Fischer (1993) published nine core principles for *Sanfte Chemie* [68]. Both dealt, among other things, with safe synthesis methods, life‐cycle analyses, the preservation of complexity, and the renewability of starting materials—which goes far beyond the mere use of renewable raw materials, biotechnology, and biomimetics. In doing so, they demonstrated both the awareness and the necessity for a more comprehensive understanding that goes far beyond simple “green” approaches. In other words, they had already developed a systems‐based view of chemistry grounded in sustainability.

As noted above, several approaches developed and applied many years before the publication of the 12 Principles of Green Chemistry went far beyond them. Furthermore, it was already known at the time of their publication that products from the chemical and pharmaceutical industries synthesized in a “greener” manner are not, in themselves, more sustainable [67, 68, 69, 70, 71, 72, 73, 74, 75]. This fundamental misunderstanding and the failure to apply the connections between green chemistry and sustainability—namely, that green chemistry and sustainable chemistry differ significantly—remain very much present today. Given the knowledge already available at the time of their publication, the question remains as to why neither the green chemistry promoted by the US EPA nor the 1998 publication by Anastas and Warner incorporated a more sustainability‐oriented, systems‐based perspective. Another interesting question is why green chemistry is to be applied only on a voluntary basis and, after decades of negative experiences with environmental pollution, problems relating to raw material extraction in connection with the chemical and pharmaceutical industries and the associated social costs, as well as the difficult relation between voluntary approaches and binding legislation, have not been incorporated into legislation. This contrast is illustrated, for example, by the European Commission's binding directive on integrated pollution prevention and control, which defines “… best available techniques’ as ‘the most effective and advanced state of the art …,” not simply available technology [61].

### 3.3 Significance, Potential Misunderstandings, and (Significant) Limitations of the 12 Principles of Green Chemistry

The 12 principles of green chemistry focus on how a synthesis can be carried out in such a way that it requires less energy, produces less waste, is less hazardous, and yields less toxic chemicals. In this respect, the 12 principles of green chemistry are important for preventing environmental pollution associated with chemical and pharmaceutical products by guiding chemists and pharmacists towards more environmentally friendly processes and compounds. “Green chemistry is the application of a set of principles that reduce or eliminate the use or generation of hazardous substances in the development, manufacture and use of chemical products” [52]. However, to make better use of these 12 principles and to avoid pitfalls, misunderstandings, or rebound effects, a more detailed examination is required. Several studies have been published on the general limitations of the 12 principles [43, 75]. The term “green pharmacy” has traditionally been used to refer to the use of natural medicines such as herbs. In the context provided here, however, the issues relating to green pharmacy beyond this are the same as those in green chemistry. The following section therefore outlines the limitations and misunderstandings regarding the application of the 12 principles themselves.

Had the 12 principles been presented in line with the life cycle of a chemical, that is, beginning with the design of chemicals, including questions of use and end‐of‐life, followed by the selection of resources and finally the planning and execution of the synthesis as the last step, a more holistic approach would have emerged. This would also promote life cycle and systems thinking and prevent the situation where, when classifying a synthesis or a chemical as “green,” often only a single principle is applied. An assessment based on all 12 principles for the synthesis of a product would draw attention to conflicts between some of the principles. This would promote life cycle and sustainability thinking, including a better understanding of why a product is more environmentally friendly than others—or why it is not—and what implications these findings would have, for example, how a compound and its synthesis need to be made more environmentally friendly. This would help to avoid greenwashing or even sustainability washing.

Some of the 12 principles are indeed guiding principles, while others are merely tools for implementing specific principles. Principle 1 deals with waste prevention. Principle 2 *2* refers to atom economy [62, 76], which is a metric, that is, not a principle that helps to compare the amount of waste generated (“how was it done”) but not the principle of waste prevention itself (“what needs to be done”). Furthermore, atom economy is limited in its quantitative aspect, as it only takes into account the atoms of the starting materials and the synthesis product: solvents or other auxiliary materials or chemicals required for the reaction, processing, or purification to isolate and purify the desired synthesis products are not included. The E‐factor, introduced as early as 1992 by R. Sheldon—long before the publication the 12 principles—is a far more comprehensive metric and a tool for waste prevention, as it defines waste as “everything except the desired product” [77]. Atom economy and the E‐factor are quantitative, weight‐based metrics. They do not consider qualitative aspects such as the toxicity of the waste produced. The need for significantly more sustainable and life‐cycle‐oriented metrics has recently been highlighted [78]. As the E‐factor shows, the synthesis of pharmaceuticals generates significantly more waste per kilogram of product than the synthesis of bulk or fine chemicals [50, 77]. In the 1990s and early 2000s, the pharmaceutical industry reduced the volume and toxicity of solvents and optimized the synthesis steps required for a given molecule to reduce waste volumes. The pharmaceutical industry also developed guidelines for selecting less toxic solvents, which enabled cost savings, for example, thanks to lower safety requirements or disposal costs. In other words, the driver for this greening was the rising costs of waste disposal and safety measures. The prevention of environmental pollution associated with pharmaceutical products, such as the presence of pharmaceutically active ingredients or excipients in the environment, was not addressed, as it did not involve any costs. To recover waste, its components can be separated and processed. They can then be reused, for example, as starting materials or solvents in the same reaction or in the synthesis of another product. However, if the chemicals have similar properties, the separation and processing of the waste components are labor‐intensive—that is, waste‐ and energy‐intensive, technically demanding, costly and time‐consuming.

Principles 3 and 4 both deal with safer chemicals through hazard reduction. While this is important, one must ask which hazardous properties of a chemical are of interest. Chemists tend to focus solely on acute and chronic (eco)toxicity. Nowadays, however, the focus is increasingly shifting to the subtle neurological effects or behavioral changes in humans and environmental organisms, that is, effects at the population level are increasingly coming into focus. These are associated with significantly longer timescales before an effect can be “definitively” attributed. Therefore, general precautionary measures and the complete absence of hazards (see Chapter 5.2) would be the appropriate goal, rather than merely their reduction. Due to the large number of different chemicals (high chemical diversity, see above), the wide range of endpoints, and the numerous different organisms, comprehensive testing is practically impossible. Therefore, a general reduction in the use of chemicals is also essential. This is not required by the 12 principles, as they focus only on individual chemicals.

Principle 5 is a subcategory of Principles 3 and 4 and a tool for applying Principle 1. Principle 6 recommends a design focused on efficiency. However, an excessive focus on efficiency may require the use of high‐value chemicals and, in the case of more efficient and effective molecules, many additional reaction steps to produce more complex chemicals, which in turn requires more energy and leads to more waste.

In accordance with Principle 7, green chemistry advocates the use of renewable resources. Anastas and Warner note that the time required for renewal “should be considered in the context of a human lifespan” [62]. Whatever this may mean, the timeframe for renewal could span several decades or even centuries, if it takes place at all. It would have been better to state that the rate of consumption should be lower than the rate of regeneration [79]. Furthermore, both biomass and recycling (see below) are subject to significant constraints as feedstocks for chemical synthesis. The cultivation and harvesting of bioresources require energy, water and chemicals such as fertilizers and pesticides. The latter, however, require additional resources and cause environmental pollution. Furthermore, monocultures lead to a loss of biodiversity, while the land used is often also needed as habitat, arable land for food production, or for building and infrastructure. The result is often a shift of problems from one area to another, for example, from energy or resource scarcity to water pollution; into the social sphere, by depriving the local population of resources; and/or into the future, when associated problems may only become apparent later. Often, the extraction of raw materials takes place in a different location from the synthesis, manufacture, use, sale, and generation of profit. One alternative is to use agricultural residues as a resource for organic chemicals. However, if such residues are removed on too large a scale or too quickly, this can have negative effects on soil fertility and biodiversity. For example, the removal of straw means the loss of the material that keeps the soil's pores open. Residues contribute to soil fertility or can be used by local communities for other purposes, such as securing livelihoods. In any case, the extraction and processing of chemicals from biological resources, even if they are agricultural wastes, also require auxiliary materials and energy, which in turn generates further waste. This can mean that the synthesis of a chemical such as ethylene using fossil oil as the feedstock is more environmentally friendly than one based on wood or lignocellulosic waste (see below) [80].

It is often assumed that biotechnology is inherently “green,” without taking a closer look at factors such as its energy requirements, the challenges involved in isolating synthetic products from water, and the associated waste generation and energy consumption [81, 82]. Another example is the “green” synthesis of nanoparticles using plant leaves or biological waste as reducing agents, without considering the extraction, for example, of the metals or the potential toxicity of nanoparticles to environmental organisms or humans, including in the synthesis process. In the technical conversion of biomass into charcoal (“bio”char), the investigation and assessment of potentially formed highly toxic pollutants such as PAHs or chlorinated dioxins and furans is often neglected. Their use as supposed *terra preta* then leads to contamination of soils and foodstuff. Recycling is one option, but it should not be assumed that it is not necessarily environmentally friendly or waste‐free (see Chapter 5). Renewable energies require many materials, including metals, which are non‐renewable. The production of cement for concrete used in dams for hydroelectric power stations is an energy‐intensive process that releases large quantities of CO_2_. Problems associated with large dams include soil salinization in surrounding areas and social tensions. It should be noted that low CO_2_ emissions are often accompanied by high metal demand (e.g., for wind turbines, photovoltaics, and electronics). The mining, extraction, and refining of metals, for example, involve high water, energy, and chemical consumption and often cause severe environmental pollution and toxic effects on the local population.

Principles 8 (unnecessary derivatization) and 9 (selective catalysis) are tools, not principles, which help to fulfill Principles 1 and 6 [83]. A metal catalyst could be more environmentally friendly than an organic catalyst. However, the use of metals (e.g., as catalysts) is, at least formally, at odds with Principle 7 of green chemistry. Organic catalysts are not inherently less toxic than metals. The use of enzymes in the aqueous phase presents its own challenges [81, 82].

Principle 10 is a subcategory of Principle 4, that is, it relates to the environment. It calls for chemicals to be degradable into harmless products, for example, to prevent their persistence in the environment. A clearer formulation, which would avoid misunderstandings, false expectations, and loopholes, would have to aim for complete mineralization into inorganic compounds such as CO_2_ and H_2_O. Incomplete degradation of chemicals in the environment can lead to persistent, often unknown, and sometimes even more dangerous chemicals (transformation products, TPs). Many known TPs of organic chemicals that arise in the environment are more toxic and persistent than the corresponding parent compounds [84]. The term “harmless” [62] can only refer to the current state of knowledge. Forty years ago, no one would have tested chemical substances for endocrine effects, let alone for neurotoxicity and other emerging toxicity endpoints. Chemicals or products of incomplete decomposition with these properties would have been classified as harmless. In most cases, we have very little knowledge about them [84]. Environmental chemistry was and remains the key sub‐discipline for identifying such contamination and the associated poisoning of food and organisms, including humans. In most cases, however, we do not even know the (structural) formulas of the undesirable by‐products of a synthesis or the environmental transformation products, let alone their occurrence or whereabouts and their effects in the environment. How, then, can we analyze and quantify them? How can we gain insights into their properties and toxicity? They are often not commercially available and must therefore be specifically synthesized to assess the associated risks to humans and the environment. Given the multitude of starting materials and the even greater number of resulting TPs, this is not feasible for either financial or time‐related reasons. Furthermore, the history of (eco‐)toxicology teaches us that we do not know what effects might become apparent in the future. In other words: a chemical or its TP may be classified as non‐toxic today, but the opposite could turn out to be true tomorrow—only after the production volume of such a chemical has risen sharply and the chemical is ubiquitous in the environment or even present in food and in humans. Incomplete mineralization is therefore not a pollution‐preventing or green measure. This applies only to complete mineralization in the environment. Early examples include chlorofluorocarbons (CFCs) and their breakdown products in the stratosphere, highly chlorinated pesticides, brominated flame retardants, and perfluorinated compounds, to name but a few. CFCs are very stable (persistent) and, under conditions of use, have low toxicity to humans. However, due to their high volatility, they escape into the stratosphere, where different conditions prevail. High‐energy UV radiation breaks C‐Cl bonds, resulting in fluorocarbon radicals and chlorine radicals as breakdown products. These radicals then form part of a catalytic cycle that destroys the ozone layer. However, this was already known before production rose sharply in the 1970s and 1980s [20]. Organic fluorine compounds and SF_6_ were detected in the atmosphere as early as 1970 [85]. Furthermore, it has recently been demonstrated that CFCs were present in the atmosphere as early as 1951 [86]. If a chemical product, such as a solvent or other auxiliary agent, is reused repeatedly and remains within a company or industry where it can be easily recycled, a design aimed at high stability is arguably more environmentally friendly and possibly even more sustainable, as it saves resources and energy and reduces waste by eliminating the need for new synthesis or recycling.

Finally, it is noted that the 12 principles should be applied “wherever technically and economically practicable” [62]. Such an unambitious target offers little incentive for improvement, as it refers to what already exists and is currently paying off, rather than focusing on what is required to avoid long‐term costs and generate future revenue through innovation. In contrast, the EU Directive calls for the best available technology, which may not be economically “feasible” at the time of its introduction but will be later once it has become more established and its application becomes mandatory [61].

In summary, while the EC Directive and the 12 principles of green chemistry are certainly important, they also have significant limitations. Products synthesised in accordance with them are not necessarily more environmentally friendly or more sustainable, as they neither take into account the fundamental principles of sustainability nor incorporate systems thinking. This applies in particular to the 12 principles of green chemistry and their lack of comprehensiveness and ambition. Future adjustments and the implementation of the 12 principles should consider the proposed improvements and clarifications.

### 3.4 Greener Versus More Sustainable Chemistry

Green chemistry is merely a technological approach [43]. Social scientists hypothesized that “all synthetic chemists could identify with at least one principle to “green” their work, which is one of the main causes for their rise” [43]. This is clearly reflected in publications on the green synthesis of chemicals. For example, thousands of publications claim that a synthesis or a product is “green” by referring to just one or two of the principles, without further explanation and often without presenting the metrics used for assessment. This prevents a more holistic view and often leads to rebound effects. It underscores the need for a far more comprehensive assessment of whether a process or product is more environmentally friendly or even more sustainable, rather than exaggerated publications that use the term “green” unjustifiably or—worse still—equate “green” or “green and sustainable” or “green chemistry” or “green pharmacy” with “sustainable.” Furthermore, it is impossible to know whether a product, synthesis, or solvent is green in an absolute sense, as this is not a given by nature. We can only judge whether it is greener than alternatives based on our human values (see Figure 2 and Chapter 2) and the application of comparative metrics.

An assessment of environmental friendliness, circularity, and sustainability requires detailed background information on why and to what extent a process or product is more environmentally friendly, more circular, or more sustainable than another. The choice of metrics and the evaluation of the information derived from them are also based on our preferences and values, which are reflected in the system boundaries set for the assessment and the criteria used. This must be made clear, and the methods, assumptions, and data used must be accessible, understandable, and transparent.

When processes are being, compromises are sometimes necessary. However, these compromises should be seen as an opportunity to gain new insights and new (business) opportunities, rather than being glossed over or used as an excuse for not making any changes. This is not only in the economic interests of companies, but is also a matter of credibility vis‐à‐vis customers, the public, and policymakers. The use of a water‐based process instead of one based on organic solvents can drastically increase the energy requirements of the process (e.g., for heating a solution) or the energy and auxiliaries required for isolating the reaction product and purifying it, particularly if the isolates are highly water‐soluble. The use of a renewable raw material such as straw or wood as a starting material, that is, the polar polymer cellulose (or hemicellulose or lignin), can lead to significantly more waste and a much higher energy requirement if, for example, a non‐polar compound such as ethylene is synthesized from it: (hemi)cellulose must be extracted, OH groups must be removed, the chain must be broken down into its building blocks (depolymerization followed by glucose extraction), the ring must be opened, oxygen must be removed again, and the C_6_‐unit must be converted into three C_2_‐units; finally, desaturation is required. Each step requires energy, auxiliary materials, and other resources and generates waste. In other words, to obtain a small, nonpolar molecule from a natural polar polymer, many more reaction steps are required compared to using nonpolar naphtha as a starting material for ethylene. Synthesis from fossil oils as a resource might be the better option in this case [80]. Since the polymer (polyethylene) must be collected anyway for reasons of environmental pollution and recycling, the limited availability of fossil raw materials is not of such great significance.

The synthesis of methane from CO_2_ and H_2_O (“Power to X”) requires a great deal of energy: CO_2_ and H_2_O are feedstocks with very low energy content (thermodynamics). This is why they are the end products of the oxidation of organic matter, for example, through combustion. Furthermore, CO_2_ and H_2_O must be activated for the reaction (kinetics). In the best‐case scenario, a catalyst reduces the activation energy but does not alter the thermodynamics, that is, the energy required to achieve the high energy content of methane or methanol. Only around 10% of the energy invested in producing methane can be used for locomotion. It would be far better to use renewable electricity directly for locomotion, for example, electrical cars. The use of hydrogen in steel and cement production or chemical synthesis would save far more CO_2_ emissions than if hydrogen were wasted on cars or even for heating. A carbon footprint analysis showed that global production of organic chemicals accounts for far less than 0.1% of the chemical industry's CO_2_ emissions [87]. Furthermore, renewable energy also requires resources (see above) and will generate recycling needs and waste in the future. Moreover, from the outset, green chemistry has prioritized economic considerations over ecological ones [43]. Neither social nor ethical aspects have been considered. In other words, green chemistry is not about sustainability. “By ignoring the fact that the financial burden of health and environmental effects is not carried by the polluting companies themselves but by individuals or the public sector, GC [i.e. green chemistry, the authors note] turns back into traditional chemistry, as shaped by the economic system that helped create it” [43]. And “… we can understand GC as a legitimation tool, born from the EPA's need to … respond to its controversial chemicals policy” [43]. This is another reason why we must draw a strict distinction between green and sustainable chemistry and pharmacy and avoid phrases such as “green and sustainable chemistry” or “green and sustainable pharmacy,” expressions that have become very popular among chemists and pharmacists.

More than 15 years after it was first outlined, sustainable pharmacy is still in its infancy [35]. More recently, more specific aspects of sustainability in pharmacy have been explored in greater detail [36]. The ACS Green Chemistry Institute defines the term “sustainability” as follows: “The Roundtable is a forum where global pharmaceutical and allied industries collaborate to advance the sustainability of manufacturing medicines by implementing green chemistry & engineering” [88]. In other words, the focus is exclusively on green synthesis and technology, as well as other topics in green chemistry, but not on sustainability; that is, on green pharmacy, but not on sustainable pharmacy. This is reflected in the subpages, which list exclusively topics related to green chemistry. There does not appear to be a Wikipedia page for either green pharmacy or sustainable pharmacy as yet. The first summer school on the topic of sustainable pharmacy was organized by Michael Müller in Freiburg (Germany) in 2022 and addressed green and sustainable chemistry, as well as other sustainability topics relevant to sustainable pharmacy, in a holistic manner.

## 4 Chemistry and the Circular Economy

### 4.1 General Background

The reuse and repurposing of substances, materials, and products at the end of their life as a resource (“waste”) for new products (“recycling”) has been a constant feature of human history, helping to manage limited material and energy resources. The use of manure and human excrement as fertilizer went hand in hand with settlement and agriculture. Composting, refurbishment, and repair were practiced from the very beginning. The reshaping and recycling of metal objects was already widespread in India, China, Mesopotamia, and ancient Egypt during the Copper, Bronze, and Iron Ages. This was based on knowledge of where the objects in question were located, their appreciation, value, origin, and their properties relevant to their composition. The reuse and recycling of paper, textiles, and glass were common practices until the recent past. The necessary knowledge was available and passed on.

Deposit systems and leasing models were introduced many decades ago, initially in response to scarce and expensive resources and later more to prevent environmental pollution than for the purpose of conserving resources. In the chemical industry, for example, there was a shift from coal to fossil oil, both of which served as sources of carbon and energy [89]. This had an enormous impact on the entire sector: Chemical and pharmaceutical production rose sharply with the growing availability of fossil oil. The easy availability of energy made it possible to ignore the finite nature of resources for a time [90, 91, 92, 93]. Still today, the global production and use of a wide variety of products continue to rise steadily [1, 9]. This is reflected in a sharp rise in resource consumption and the associated environmental pollution [1, 5, 6, 7, 8]. Thus, humanity's material consumption has increased in an unprecedented manner over the last 50–70 years, both in qualitative and quantitative terms, as well as in terms of time and space [1, 5, 6, 7, 8].

From the very beginning, the chemical industry has used unwanted reaction by‐products as raw materials or as components for other products, as was already the case in prehistoric times (e.g., birch tar as a sealant): Coal tar, a residue from town gas production, was used as a starting material for the manufacture of synthetic dyes, which formed the origins of the modern organic chemical and pharmaceutical industries. Such cascade utilization has long been practiced in the chemical industry and remains common today in companies or at so‐called integrated sites (Chemiepark). In fact, this practice utilizes more or less well‐characterized waste from synthesis and offers financial benefits. All of this was later supported by the fact that the disposal of chemical waste became costly.

The general concept of a modern circular economy was first developed as early as 1971 [94]. In 1982, it was further elaborated in detail, including all its fundamental principles and aspects [95, 96]. This took place more than 25 years before it was “reinvented” [97]. In the 1980s, Stahel referred to it as “Back to Cradle” and “Cradle to Cradle,” with the latter term also being adopted and promoted by others at the time [98, 99]. The terms “health” and “nutrient” were used in this context by some authors [98, 99]. However, nature knows no such value‐related connotations, and even from an anthropocentric perspective, these are only vaguely defined.

The driving force behind the circular economy today is the renewed realization that resources—including a clean environment—are finite, a fact that is also reflected in the concept of PBs. This is currently most evident, for example, in the case of metals (particularly rare earth elements and other so‐called strategic or technology metals), phosphate, graphite, and fossil raw materials, as well as many other critical resources [100, 101, 102, 103]. At the same time, there is a desire to avoid waste and environmental pollution caused by products [104, 105]. In this context, the circular economy is also an important concept for the chemical and pharmaceutical industry. Nowadays, this is often understood to mean transforming a linear system into a circular one, which leaves the unspoken expectation that “all” product‐related waste and resource problems will be solved. In reality, however, this is not the case [103, 104]. In modern products, various elements are intermingled at all levels—in chemicals, materials, and building blocks, for example, in metal‐organic frameworks (MOFs), plastics, fibers, electronic components, alloys, and photovoltaic materials. Products such as textiles, furniture, packaging, computers, mobile phones, bicycles, cars, personal care products, medicines, and buildings are made up of these. The products and their components intermingle within economies during their use and at the end of their life cycle, for example, in waste streams at local, regional, and global levels. Others, such as personal care products, pharmaceuticals, disinfectants, pesticides, and detergents, enter the environment at the end of their life due to their use. Others end up in the environment unintentionally but are also linked to their use, for example, through the release of volatile constituents and abrasions from surfaces such as façades, textiles, or tires. All these cannot be recycled. In a circular economy, just as in a linear flow economy, the variety and quantity of products increase, as does resource consumption [1, 9, 105, 106]. The service life and lifespan of products, as well as their significance for the circulation and the availability of products for recycling, are often not considered. Mainstream descriptions of a circular economy tend to refer to nature's material cycles, for example, for organic materials such as leaves or wood, without further mentioning the serious inherent limitations of the circular economy [97, 98, 99, 107, 108]. Such an analogy neglects fundamental laws of nature, such as the laws of thermodynamics (see Chapter 4.4) [105].

### 4.2 Circular Chemistry

The need to take circularity into account when designing chemical processes and products is receiving increasing attention [108]. Keijer and colleagues presented 12 principles of circular chemistry (Table 1) [109]. Six of the 12 principles of the circular chemistry closely resemble the 12 principles of Green Chemistry (see Table 1); others resemble the principles of “Greener Engineering.” Still others relate to the recycling ladder and recognize, in particular, the continued use of products as a central aspect [110, 111, 112]. A more detailed hierarchy within technical recycling itself has recently been proposed [113]. Keijer and colleagues argue that aligning the chemical industry with the 12 principles of green chemistry may lead to unsustainable practices in chemistry, as the 12 principles of green chemistry do not take the circular economy into account.

Like the 12 principles of green chemistry, the principles of circular chemistry also aim to produce more environmentally friendly materials and products, but within the framework of a circular economy. Like green chemistry, the concept of circular chemistry focuses solely on individual chemicals, materials, and products [109, 114, 115]. The 12 principles of circular chemistry neither address the challenge of ever‐increasing flows of substances, materials, and products, nor do they tackle genuine sustainability issues. They neglect the systemic consideration of such flows and energy at local, regional, and global levels, as well as the challenge posed by the ever‐increasing diversity of materials and their dynamics in mixed products for recycling. For example, the role of downstream users and the design of the associated material flows beyond individual materials and products—including the impact of design on the collectability for recycling at the end of a product's useful life—are not considered. Certain products of the pharmaceutical industry, that is, the medicines themselves and their excreted metabolites, cannot be kept in the cycle. However, many materials and products, such as pharmaceutical packaging, could be recycled, which makes circular chemistry important for the pharmaceutical sector as well.

### 4.3 Natural and Human‐Made Material Cycles

Over millions of years, the natural world adapts by utilizing its materials for as long as necessary and subsequently reusing the building blocks from which they are formed. Circular chemistry and the circular economy refer to nature's closed material cycles and its seemingly inexhaustible supply of energy. However, nature's cycles differ greatly from those in the technosphere. Only a few chemical elements are used in nature in significant quantities across all levels and circulate globally (e.g., carbon, hydrogen, oxygen, nitrogen, and, to a far lesser extent, sulfur and phosphorus), if one disregards geological timescales. Most of these elements circulate globally only in the form of simple inorganic compounds. The interconnected cycles are in a well‐balanced global steady state, which has been established by nature over long periods of time on a global scale and today exhibits a stable condition with very minor and only very local imbalances. Humanity has significantly disrupted these cycles in recent decades, for example, through the increasing release of CO_2_ and methane; the increasing mobilization of phosphate, nitrogen (as nitrate), and metals; the production of plastics; and the introduction of many other novel substances. It is not only a question of quantity, but also a question of chemical diversity. As mentioned above, all non‐radioactive elements and even some radioactive elements are now used in considerable quantities and distributed by humans worldwide, although for most this only began in the last 50 to 100 years [8]. Rare‐earth metals, for example, have only been mobilized by humans in significant quantities in recent decades. In nature, the proportion of mobile rare‐earth metal species is very low. Metals, apart from gold and some silver and copper, do not occur in nature in elemental form. The vast majority of metals are immobilized in the form of minerals and are scarcely accessible to living organisms; they occur, if at all, only in low concentrations in organisms, despite their enormous importance to them. A similar example would be the metalloids. Halogens, as a functional group of organic compounds, are present in organisms, if at all, only in low concentrations within small molecules. Iodine, for example, occurs only in very low concentrations as a component of certain human hormones or in the pigment of purple sea snails. Organic fluorine, chlorine, and bromine compounds, as well as inorganic bromine, play no significant role in life. The concentration of their organic compounds outside organisms is also very low, mainly as derivatives of methane.

Today, between 150 000 and over 300 000 non‐natural chemicals (“novel substances”) make up the diverse mixed streams of organic synthetic substances in the technosphere. Some of these are components of complex materials, building blocks, and products that are entirely foreign to nature, including living organisms (“xenobiotics”). In nature, there are only a few organic mass chemicals and building blocks, and the bulk materials produced from them, namely fatty acids, isoprenoids, sugars (glucose, starch, chitin), a limited number (20) of amino acids (proteins), and phenols (e.g., lignin and other polyphenols). The building blocks of these materials are reused (“recycled”) very locally on a scale of a few meters or less or, in many cases, literally at the very spot where they were formed. Furthermore, these natural products and the associated material flows have evolved together over a long period of time. There were no significant short‐term changes on a larger spatial scale. Life, as an important component of the system, was therefore able to adapt in the long term to the newly formed chemicals and materials or indeed could only have emerged because the basic building blocks (e.g., amino acids) were already present. This stands in stark contrast to the regional and global short‐term, rapid changes in the cycles of elements, molecules, materials, and products caused by human activities. The climate change, eutrophication, and acidification we are currently experiencing demonstrate how even a small change of concentrations can have dramatic consequences within a short period of time (see also Chapter 4.5).

### 4.4 Thermodynamics

#### 4.4.1 Insurmountable Limits and the Illusion of Zero Waste

For example, Keijer et al. claim that the application of circular chemistry would lead to a waste‐free industry [109]. Others tacitly assume this when they speak of recycling. However, zero waste is impossible according to the three laws of thermodynamics. They can be summarized as follows: [116] (1) One can never win; one can only break even. (2) One can only break even under perfect conditions. (3) One cannot reach perfect conditions.

Waste must be collected, products dismantled, and components separated and then treated (chemically). The efficiency of the entire process cannot exceed that of the least efficient of these steps. If, for example, waste collection is only 30% efficient, the overall recycling rate cannot exceed 30%. Even if a recycling process consists of just four steps—collection, dismantling, physical separation, and chemical treatment—and all processes operate at 95% efficiency, 19% is still lost. On top of this, technical processes operating at 95% efficiency are very rare. Another fundamental limitation of recycling is that products such as many pesticides used in the open environment cannot be returned to the cycle or recycled at all. The same applies to products that inevitably end up in the environment after use, for example, disinfectants, medicines, detergents, and personal care products. In addition, abrasion and leaching from material surfaces, facades, tires, casings, etc. cannot be returned to the cycle or recycled at all.

To compensate for the loss of quality during the use phase or recycling processes, substances resulting from aging processes must be removed and fresh material added, e.g. new fibers in textiles and paper, and new (base) metals and doping elements in alloys. These must first be extracted and purified or synthesized, respectively. The closer one wishes to get to perfect conditions, the more energy is required to maintain the state achieved. This also applies to maintaining highly ordered non‐equilibrium structures such as life, societies, or products [117]. More or less complete recycling of a car might be possible in the future [118]. However, this does not mean that there will be no more waste, as both material and energy inputs will be required. The recycling of more complex products (and waste) requires more steps and more energy than for products of lower complexity and waste streams of lower diversity. In other words, the increasing diversity of products and materials in turn leads to a rise in energy and material entropy: The enormous increase in diversity and complexity contributes to the multitude of microstates (W) within the technosphere and, according to Boltzmann's famous formula, *S* = *k* ****ln *W*, leads to an increase in entropy. The effects of this are evident in a rising demand for energy, auxiliary materials, and processes (e.g., separation); in waste generation; in dissipative losses; and in the creation of NIAS throughout the life cycle as well as within the recycling process. The increased technical effort also affects economic viability, which often results in products not being recycled and their constituents being lost as waste (dissipation).

Another form of increasing entropy is the growing loss of the information required for the collection and (re)cycling of end‐of‐life products or waste, that is, to know where they are and what they consist of. If this information is not available, they can neither be collected nor efficiently recycled. If the composition is unknown, efforts are required to obtain this information (e.g. through physical or chemical analysis or internet research), which will add to incomplete separation and higher recycling costs.

If a zero‐waste target is to be pursued, it is often forgotten that the closer we aim to get to this target in a technical process, the more it will cost (in terms of energy, resources, money, the generation of other waste, etc.), and rebound effects will increase. In any case, 100 per cent recycling is impossible, as this would require infinite energy [119, 120, 121, 122, 123]. Ultimately, we cannot avoid the increase in entropy. Physicists, chemists, and pharmacists know this and are educated in thermodynamics. They therefore share responsibility for preventing potential misunderstandings among the public, economists, or politicians: the increase in entropy—that is, the loss of usable material and energy, waste generation, and environmental pollution—is inevitable! [119, 120, 121, 122, 123]

According to thermodynamics in the long term, the breakdown of ordered structures can only be delayed, not avoided. This delay comes at a price and can only be achieved for a certain period through the continuous input of energy. As soon as we stop the energy supply, the entropy of a system increases and the ordered structure collapses [117]. This is one of the reasons why we die when we stop eating and why, as long as we eat, we excrete entropy‐rich material. The more we “combat” entropy, the more entropy will ultimately arise, albeit in a different place or in the future, but ultimately, unavoidably. Even if infinite energy were available to combat entropy, that is, dissipative losses and the generation of waste, this would lead to an infinite increase in entropy, the combating of which would in turn require infinite energy, and so on. We can reduce entropy on earth locally for a certain period of time by investing energy that we obtain from the sun. The sun does not send a bill. But the intermediary “actors” (e.g., the products needed to “harvest” solar energy) must be paid for in various forms of entropy. Unlike energetic entropy, the earth cannot get rid of material entropy [121].

The more energy and material we consume and mobilize, the greater the expected entropic losses of energy and matter. The same applies to industrial systems and their products. Ultimately, everything becomes waste—that is, matter “lost” for further use (dissipation)—including the deterioration of products and structures over time [123, 124, 125]. Dissipative losses, that is, the increasing entropy of energy and matter, are evident, for example, in the incompleteness of collection and the deterioration in the quality of materials. The latter is evident, amongst other things, in the inevitable incomplete separation of products and their components for and during recycling. However, chemical reactions during the use of products (“aging”) and during recycling, which lead to undesirable by‐products and degradation products, are also unavoidable and contribute to the increase in entropy [119, 120, 121, 122, 123]. These losses also include the unintended dispersion of metals as a result of their use, that is, an unavoidable decrease in concentration, which manifests itself, amongst other things, in their distribution in (very) low concentrations throughout the entire life cycle over increasing space, for example due to abrasion and oxidation during use; losses during recycling, through catalysis; or because they end up in slag [121, 123, 124, 125]. Although energy and matter are not lost in the physical sense, they are no longer available for use for economic, technical, or chemical reasons.

#### 4.4.2 Chemical Diversity, Product Complexity, and Dissipative Material Losses

The concepts outlined above generally consider only individual products. They exhibit increasing diversity and complexity, from the atomic and molecular level right up to the material, component, and product levels. The individual products and their constituents accumulate in an economy to form total material flows at local, regional, and global levels, whilst simultaneously intermingling. This mixing process is also subject to temporal dynamics: the type, diversity, and proportions of the components change over time, often at different rates—for example, in production on the one hand and recycling on the other—as well as in different locations and at different times. This increases the inevitable dissipative losses throughout the life cycle of products, including recycling itself.

Therefore, design at the atomic and molecular level of materials, building blocks, and products including the material flows resulting from the various spatial and temporal scales, which have hitherto been scarcely considered in this context, are of central importance for recycling [109, 114, 115]. For this reason, it is also necessary, on the one hand, to understand what degree of purity is sufficient for a specific application of recycled products, in line with the intended use, rather than always demanding the purest and highest‐quality raw materials and/or recycled products. On the other hand, however, this may, in the long term, be accompanied by further undesirable deterioration and contamination of substance, material, and product flows. In other words: it will not eliminate the fundamental limitations of a circular economy. To fail to recognize this would be wishful thinking that denies physical reality. This highlights the need to reduce the volume, diversity, complexity, and fluctuations of substance, material, and product flows in space and time. The aim is not to maximize a flow economy through recycling in the conventional sense, but to optimize it from a systems perspective.

#### 4.4.3 The Illusion of Upcycling

The term “upcycling” suggests that recycling is possible indefinitely. This is yet another attempt to deny the laws of thermodynamics: Firstly, so‐called upcycling often merely results in the addition of just one further cycle of use. A second cycle is often not possible, as new additives are added to the recycled materials to impart the properties required for the new application, and the materials are of inferior quality after recycling due to aging during the recycling process. That is, their chemical composition changes even during use and in the recycling process itself, for example, due to NIAS. The recycled material can no longer be used for its original purpose or only after considerable effort to upgrade it. One such example of “downcycling” is the polymer polyethylene terephthalate (PET), which is of very high quality when first used for water or soft drink bottles, while after the plastic bottles have been recycled, it serves as a textile fiber. However, the textile requires the addition of other chemicals and is contaminated by NIAS, which reduces its purity.

Any upgrading of materials involves energy consumption and results in waste, energy losses, and material losses! According to the laws of thermodynamics, these losses are unavoidable. And even from the perspective of a material‐ and energy‐based recycling system, there can only be downcycling. To speak of “upcycling” merely suggests that the system boundaries of the assessment have been set too narrowly as for energy and materials. It ignores the fact that the dissipative energy, waste, and environmental impact caused by recycling, as well as the effects of the raw materials and auxiliary substances required, will inevitably come to bear elsewhere or in the future, as will the associated costs of addressing these problems. In other words, there is neither upcycling nor endless recycling and it is certainly not cost‐free when all technical and economic aspects are considered. Furthermore, economic recycling is only possible for a fraction of a waste material or product. Recycling also generates waste that needs to be managed. Therefore, in such cases, the term “(re‐)valorization of part of the waste” should be used instead of “upcycling.” The limitations of today's existing systems and infrastructures can certainly be improved through new, more suitable, effective, and efficient systems [118, 126, 127], including the collection of obsolete products. However, in the long term, we will not be able to overcome thermodynamics and the clear limits it imposes.

#### 4.4.4 How Can Dissipative Losses be Reduced?

Thus, if the three laws of thermodynamics tell us that we cannot win and cannot even achieve a balanced outcome, we must strive to lose as little as possible. To do this, we must first and foremost not only reduce the scale and the temporal‐spatial dynamics of material, substance, and product flows, but also their complexity, including the diversity of their constituents and their spatial‐temporal dynamics, to keep dissipative losses as low as possible. We must acknowledge the fundamental laws of chemistry and physics and their significance for the fundamental and practical limits of the circular economy and recycling outlined above. We must therefore focus on recycling at all levels—not only from the atomic to the product level and across the entire life cycle, but also on the overarching material and product flows—and must not forget thermodynamics and thus the physical and chemical fundamentals. To minimize losses, chemical‐based recycling should not be the first step but the last one (Box 1). Furthermore, we must rethink what level of quality is required for a specific product and its application—the quality must be fit for purpose, but no more than that. Achieving higher performance with less material of lower diversity and complexity is both a challenge and an enrichment for the chemists and pharmacists of the future. The task is to optimize, but not maximize, product performance within the context of the system.

**BOX 1: Hierarchy of approaches to recycling [113]**

1. Minimize unavoidable losses; recognize that high‐quality recycled products require the addition of new material; avoid shifting problems into the future or to another location or environmental medium; reuse as much as possible of the waste generated in the recycling process (“cascade recycling”); distinguish between optimum and maximum in systems thinking.

2. Retain parts (“macroscopic” building blocks).

3. Maintain shape and size (e.g., do not cut materials such as plastics, textiles, sheet metal, and parts, as well as electronic components, into pieces).

4. Preserve the material (e.g., reshape thermoplastics, metals and alloys; avoid thermosets as they cannot be reshaped).

5. Preserve composition (e.g., during reprocessing, do not add new constituents such as additives or other components, and do not remove any).

6. Preserve molecular building blocks (e.g., solvolysis of polymers first instead of depolymerization; solvolysis can be used to remove unwanted legacy chemicals and unintentionally present substances [NIAS]).

7. Preserve molecular building blocks (e.g., monomers from depolymerization).

8. Preserve atoms (e.g., pyrolysis of plastics, separation of metals during metal recycling, and prevention of the transfer of metals into the slag; can be used to remove toxic elements).

9. Energy recovery (so‐called “thermal” recycling of organic materials, which is simply incineration, i.e., energetic downcycling, and leads to the ultimate destruction of the materials).

### 4.5 Time Ecology

Time, in its many forms and manifestations, lies at the heart of sustainability. A time‐ecological perspective enables us to take into account the significance of the lifespan of resources, processes, and products throughout their entire life cycle, as well as the associated rates of change, for example, in terms of depletion and regeneration [79, 128, 129, 130, 131]. For example, extending a product's lifespan can be beneficial if products remain in the usage cycle, that is, can be reused and repurposed multiple times [132]. However, the longevity of products and the stability of their components can pose additional challenges for recycling, as they may require greater effort for disassembly and separation, as well as for the environment due to their longevity. A long product lifespan leads to the mixing of several product generations, which increases the complexity of substance, material, and product flows and makes recycling more difficult. Therefore, design for an adapted, optimized rather than a maximized service life is required to reconcile the competing objectives of a long service life (i.e., stability under given conditions during use and thus resource conservation) and either simple recycling or rapid and complete mineralization in the environment at the end of the service life. The balance depends both on the duration of use of the products and on the service life of the products and their components, depending on the specific application of the product, the required (not the desired) service and function, and the specific conditions prevailing during the life cycle of the product and the service. For example, a medicinal product or chemical substance should be stable enough for shelf life over months or even years, but it should also be excreted from the human body within a few days and, following excretion/release into the environment, mineralize within a few days or weeks (“Benign by Design”). It has been shown that such chemicals and even active pharmaceutical ingredients can be developed [133, 134, 135, 136, 137]. We must also consider how long a component or material remains “hidden” within a product, that is, when and where the material will become available again, in what quality and quantity, and how easily and quickly. This depends on the lifespan of its application, such as when the same type of polymer is used in packaging compared to in textiles, buildings, or cars.

The waste generated during recycling, synthesis, and manufacturing often originates at a different location, leading to delays in the production and use of a particular product. These time lags must be considered and understood. Recycling takes place in a different technological, cultural, and economic environment, as well as in a different temporal context, from the synthesis, manufacturing, and use of products. Recycling facilities may not yet be available or may no longer be available, either because they have not yet been built or because they have already been decommissioned for economic reasons. If, for example, the product's lifespan is too long, the quantity available for recycling is too small, or it enters the main product stream as a contaminant or harmful substance or product. Often, many types of similar but not identical products coexist on the market, such as different types and generations of mobile phones, computers, televisions, cars, or textiles, which contain different materials in varying combinations and concentrations and components linked to different technologies. Over time, the market share of each product will change due to product innovations.

All of this contributes to the complexity of the material flows that need to be recycled. High rates of innovation may lead to problems in the future, as recycling companies may not be able to adapt their technologies in time to new products or a greater variety of products—or both. If the rate at which a chemical is released into the environment is higher than its rate of degradation, even chemicals that mineralize in the environment remain there permanently (known as “pseudo persistence”) [138]. The level ultimately reached is determined by the ratio of its release to its residence time in an environmental compartment. The same applies to products that need to be recycled. If the ratio of release of products and availability of recycling capacities is not fitting, materials are lost. So, rather than striving for maximum product lifespan and stability, we need to better understand how the lifespan of chemical products and their use are interlinked from a systems perspective: During the product design phase, it is essential that the lifespan is adapted to the social, economic, technological, and ecological context in which it is used. The frequency of innovation and the lifespan of products in the technosphere and the economic sphere must be compatible with the intrisic timescales (Eigenzeiten) and temporalities of natural and social systems, too [79, 128, 129, 130, 131].

## 5 How Chemistry and Pharmacy Can be Embedded in Sustainability

### 5.1 General Considerations

Chemistry and pharmacy have numerous links to sustainability. The following chapter sets out how chemistry and pharmacy can be embedded into sustainability. Figure 3 illustrates the relationship between chemistry and pharmacy and the SDGs and the PBs [139, 140, 141]. The PBs define the safe human operation space. The SDGs help to ensure that we remain within the PBs and take issues of justice into account [142, 143] not only with more environmentally friendly and recyclable products, but also, whenever possible, with fewer or even no products through alternative business models. These enable the reduction of substance, material, and product flows, as well as their diversity, at all levels—local, regional, and global [144, 145].

Sustainability aims to ensure that the needs of the present generation are met without compromising the ability of future generations to meet their own needs. This encompasses three overarching sustainability strategies—sufficiency, consistency, and efficiency—and takes into account all stakeholders throughout the entire life cycle of products [142, 144, 145]. Ethics is also an essential feature of sustainability and is therefore indispensable for sustainable chemistry and pharmacy [146, 147, 148, 149, 150]. Another important point is that focusing on isolated measures and subsystems often leads to undesirable side effects and rebound effects. Subsystems must therefore be interconnected. In other words, systems thinking is another constitutive element of sustainability and thus of sustainable chemistry and pharmacy, rather than isolated, piecemeal solutions [151, 152].

Already addressed in the 1987 Brundtland Report and the 1992 Rio Declaration, the 2002 UN World Summit on Sustainable Development in Johannesburg reiterated that we must replace hazardous compounds, increase resource efficiency, and work together to develop better chemicals management globally [153]. The Summit proposed the target that, by 2020, chemicals should be “used and produced in ways that minimize significant adverse impacts on human health and the environment.” Over time, various international conventions addressing chemicals have come into force, such as the Montreal Protocol on Substances that Deplete the Ozone Layer, the Minamata Convention on Mercury, and the Stockholm Convention on Persistent Organic Pollutants (POPs). Each one deals either with a single chemical or with small groups of chemicals only. As a result, while some problems have been addressed, others persist or have emerged newly (e.g., plastics, PFAS). What is needed is a more comprehensive and integrated approach based on groups of chemicals and the products manufactured from them, rather than on individual substances [140, 143, 151, 152], including positive lists.

### 5.2 Sustainable Development Goals and Planetary Boundaries

In 2015, the UN adopted the 2030 Agenda for Sustainable Development, including the SDGs, to secure a sustainable future for us all. The SDGs address many pressing, interlinked challenges facing humanity, such as population growth, industrialization, and urbanization, food security, healthcare, clean water and sanitation, and climate change. According to the UNEP's “Green and Sustainable Chemistry” framework manual, chemistry plays a role in all 17 SDGs, including explicitly social aspects [154]. The planetary boundaries approach describes the impacts of human activities on the Earth system and the associated limits. It is linked to the WHO's “One Health” approach and the planetary health approach, for example, in relation to sustainability and consumption [155, 156]. Exceeding planetary boundaries would push the planet past a tipping point and set it on a course that is less supportive of human life or even renders it impossible. The activities of industrialized society are regarded as the main drivers of this.

As discussed above, we must recognize that humanity has produced an enormous diversity and quantity of chemicals and products and that a considerable proportion of these are not reused or have entered the environment. Within the PBs, synthetic chemicals are grouped together with genetically modified organisms (GMOs) as “novel entities.” This is because they are alien to nature and their diversity does not allow them to be named individually. It has been suggested that the planetary boundary for synthetic chemicals has already been exceeded [157]. It is important to note that we cannot truly know the planetary boundaries for chemicals, but the fact that we may be close to these boundaries or have already exceeded them provides a strong argument for precautionary measures (see below). The SDGs reflect this.

The SDGs provide guidelines on how to remain within the planetary boundaries. Almost all SDGs contain sub‐goals relating to chemistry and pharmacy. Sustainable chemistry and pharmacy are the approaches and understanding that enable chemistry and pharmacy to contribute to the SDGs in a sustainable manner. Circular chemistry and pharmacy, in turn, are the tools that help to keep chemical and pharmaceutical products within the technosphere's cycles. All the compounds needed for materials and products to be sold must be synthesized in accordance with the principles of green chemistry. Chemistry and pharmacy, as non‐normative sciences, provide the necessary knowledge for this. They must all interact with one another to support each other.

### 5.3 Precautionary Principle

The precautionary principle is a guiding principle that urges caution towards the unknown future, including the impact of innovative ideas [158, 159, 160]. The principle advocates being aware of the limits of our knowledge and actions. The significance of the precautionary principle for chemicals was already addressed in the 1992 Rio Declaration. The larger the system concerned and, consequently, the bigger the timescales, the greater the significance of the precautionary principle [128, 129, 130].

We cannot prove any positive effects, only negative consequences [158, 159]. Precaution is therefore an essential component of sustainability and must also be applied to the chemical and pharmaceutical sector, given the high diversity and ubiquitous presence of chemicals and pharmaceuticals; the spatial and temporal scales associated with their manufacture, use, and end‐of‐life; and, finally, the large quantities that enter the human environment and the wider environment, affecting biodiversity and systems. In other words, not only for reasons of sufficiency but also as a precautionary measure, the quantities and diversity of chemical substances and products must be significantly reduced. For the same reason, prevention is far better than substitution [161]. Positive lists of chemicals suitable for use would be far more effective and would support the development of more environmentally friendly, circular, and sustainable compounds, products, and business models than (single‐substance) bans, given the lengthy timeframes associated with their drafting and the challenges of finding non‐regrettable substitutes. Positive lists would offer companies much greater and more reliable long‐term guidance for designing the products of the future, rather than merely having to react to successive bans on chemicals and constantly redesigning substances.

## 6 Sustainable Chemistry and Sustainable Pharmacy

Defining the term “sustainable chemistry” has always been a challenge. It was originally coined by the German Chemical Society [57]. When the term was first used by the IUPAC and the OECD in the 1990s, it was very closely linked to green chemistry and differed, particularly on the issue of resources, from that of the US EPA (see Chapter 2) [45, 49, 55, 67, 68]. In 1994, the term “Simple Chemistry” was introduced in Japan [162]. Other definitions, more strongly focused on sustainability, were published in the late 1990s and early 2000s, emphasizing that green chemistry and sustainable chemistry are not synonyms [45, 46, 47, 55, 58, 67, 68, 163]. In 2007, according to Krasnodębski, “… a seemingly minor edit to the Wikipedia entry on green chemistry changed the beginning from ‘green chemistry is…’ to ‘green chemistry, also called sustainable chemistry, is…’ and including a redirection hyperlink connecting the two terms and presenting them as entirely equivalent. Although the definition has been updated slightly—to ‘Green chemistry, similar to sustainable chemistry or circular chemistry’—underlines the focus of green chemistry on pollution prevention, only” [45]. In this sense, many definitions of sustainable chemistry published later remained very similar to those of green chemistry. As previously discussed (see Chapter 2, 3), the concepts of green chemistry and circular chemistry are not synonymous with sustainability. This misunderstanding remains widespread in academia, industry and government bodies. Some examples among many are the description of a synthesis as “sustainable” or the call to “produce more of it” [118, 127, 164, 165, 166, 167]. The concept of sustainable chemistry was explained in more detail in UNEP's “Global Chemical Outlook II” [1]. Mutlu, and Barner, and others emphasize that green, circular, and sustainable chemistry are closely interlinked, but cannot be used synonymously [168]. Maxim points out that “Most literature relies on a narrative of its [i.e. of green chemistry, author's note] birth … through the original work by Anastas and Warner and the successful networking and institutionalising activities that followed. However, this perspective … highlights a contradiction between the … message of the founders of green chemistry, [i.e. Anastas and Warner, author's note] and their strategy of promotion, which is uncritical of ‘brown’ chemistry and excludes participation by civil society and the public” [43]. The latter, however, is constitutive of sustainability. A definition published in 2023 focuses heavily on green chemistry but also extends its scope to other socially relevant areas [169]. Although this was a step forward, no explicit reference was made to the principles of sustainability or to intangible alternative business models or similar concepts. It is steered towards viewing selling products as the most important pillar. A report published in 2023 by the US Federal Office of the White House contained a somehow more comprehensive understanding, aiming to address the circular economy and more complex products, meet societal needs, and contribute to economic resilience [170]. However, the focus *de facto* remains strongly on products and manufacturing processes. The report was no longer available on the White House website in August 2025. De Souza et al. conclude, with regard to the Federal Office's understanding, that “the way in which it [sustainable chemistry, author's note] was defined makes it clear that the definition resembles that of green chemistry” [71]. Although the recently published Stockholm Declaration appears, at first glance, to refer heavily to sustainability, it has only a very limited connection to sustainability. Instead, it largely concerns green chemistry (see, e.g., the “essential elements” of the declaration) [171]. The American Chemical Society has only recently updated its homepage and now refers to “green chemistry for sustainability,” whereas until 2024 it was simply called “green chemistry.” Although the 2024 homepage is no longer accessible, the old definition from 2024 remains available on a subpage [172]. Furthermore, “green chemistry for sustainability” does not mean developing sustainable contributions from chemistry to sustainability or even embedding chemistry within sustainability. Slootweg notes that “currently, the approaches of green chemistry, circular chemistry and SSbD [Safe and Sustainable Design, author's note] often operate in isolation from one another, which is not ideal, as each approach, although comprehensive within its scope, addresses only certain aspects of sustainability” [173]. This underscores once again that a broader understanding is required: chemistry must be embedded within sustainability.

**FIGURE 4:**
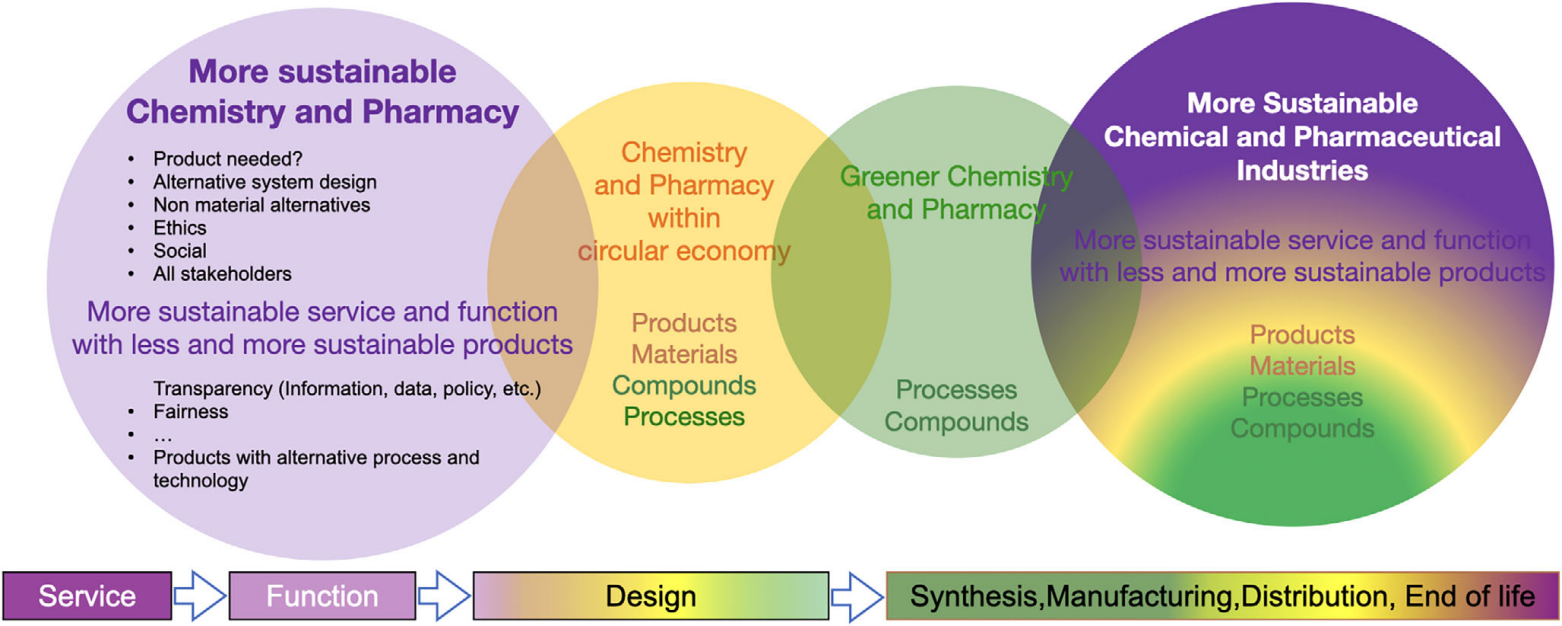
The general approach to integrating chemistry and pharmacy into sustainability: First, the services, functions, and intangible sustainability aspects are identified; then, services and products are planned accordingly, considering their end‐of‐life (circularity, including recyclability and biodegradability in the environment). Only then, following these assessments, synthesis, manufacturing, and distribution should be done, where applicable.

All of the above‐mentioned attempts to redefine green chemistry directly or indirectly and to “market” it as sustainable chemistry fail to address at all, or at least not fully, at least one of the fundamental principles of sustainability—sufficiency, efficiency, consistency, precautionary principles, systems thinking, ethics, and so forth (as presented in Figure 4, left circle)—let alone several of them. Social aspects, through at least the mention of “society” or even “ethics,” were only considered by a few. The EU's SSbD approach, which in turn is not to be equated with sustainable chemistry but is important for enabling it, provides for a social assessment during the design phase of new chemical products [174, 175, 176]. Some of the authors mentioned above advocated for the inclusion of the environment and justice, but not for justice in general. In 2025, the International Union of Pure and Applied Chemistry (IUPAC) published the Guiding Principles for Responsible Chemistry: “Chemists should act for the benefit of humanity and the planet” [177]. This is another important step forward in embedding chemistry within sustainability. However, there are still some weaknesses and inconsistencies. For example, there are eight so‐called guiding principles. The focus of these guiding principles lies exclusively on the hazardous properties of chemicals and risk reduction. Safety and risk prevention are important issues, but quite far away from sustainability. Their implementation must be a given before sustainability is even considered. The other principles, including responsible innovation, ethical conduct, inclusivity, and equity, are closer to sustainability; while they are mentioned several times, they are not elaborated upon analytically. Some of the other guiding principles include elements that could also be associated with sustainability, such as inclusivity, equity, and belonging. Integrity and accuracy, for example, are part of ethics or at least of scientific quality. The principle of communication does not necessarily refer to transparency, which is important for sustainability. As far as industry is concerned, “Responsible Care” is mentioned as a further voluntary industrial approach. A study published on the “Responsible Care” program of the German Chemical Industry Association highlighted that “the potential for opportunism can overcome the isomorphic constraints of even powerful self‐regulatory institutions, suggesting that effective industry self‐regulation is difficult to sustain without explicit sanctions” [178]. This is an ongoing criticism from many sides, which, as mentioned above, also applies to green chemistry. The impact of Responsible Care, as implemented by companies, on environmental protection does not differ significantly from other measures [178]. Another point is that this initiative focuses only on the most hazardous chemicals. Less hazardous substances and the many unknown substances and properties are not considered. Sustainability itself is not included. The principle of responsible innovation is “with a conscious focus on anticipating and mitigating harmful consequences for people and the planet” [177]. While this is important, one can only foresee what one already knows. The known unknowns and the unknown unknowns are not mentioned. However, given the high diversity, complexity, and quantities of the chemicals, pharmaceuticals, and related products used, these are likely to represent the greater present and future risks. Therefore, precaution is an important component of sustainability.

For chemistry to contribute to sustainability in a sustainable manner, it is not enough simply to refer to or pursue “chemistry and sustainability” or “chemistry for sustainability.” The same applies to pharmacy. Rather, it is crucial to understand that chemistry and pharmacy must be embedded within sustainability, form part of sustainability, and fulfill connecting functions, rather than merely being an add‐on [179]. Instead of a product‐centered approach, a service‐oriented approach is essential [132, 180]. It is simply not nearly enough to merely supply more environmentally friendly products or to facilitate the circular economy and recycling. It is absolutely essential to put sustainability first and above all else. Only then can the chemical and pharmaceutical industries contribute to sustainability in a sustainable manner. In some cases, it is even more sustainable to dispense with chemical or pharmaceutical products altogether (see below). Sustainability must be the overarching approach, that is, chemistry and pharmacy must be embedded within sustainability. Accordingly, the necessity of chemical or pharmaceutical products must first be questioned when considering the services to be provided, which is what chemistry and pharmaceuticals mean in the context of sustainability [181]. A simple definition of sustainable chemistry or pharmacy is neither desirable nor feasible, as chemistry and pharmacy, both as sciences and as industries, are multifaceted, involving numerous products and applications as well as many social, economic, and ethical aspects [182, 183].

The interconnection of chemistry and pharmacy, in all their diverse facets, with numerous disciplines such as ecology, economics, social sciences, and even philosophy (e.g., transparency, ethics, and justice), including temporal and spatial dimensions, is fundamental to embedding chemistry and pharmacy within sustainability. Such an understanding enables them to operate within the framework of the PBs and contribute to all SDGs in a sustainable manner (Figure 3). It is therefore essentially transdisciplinary. Its characteristics have recently been summarized (Box 2). In contrast to green chemistry and circular chemistry and pharmacy, sustainable chemistry and pharmacy are linked to sufficiency within the context of sustainability. As such, they are interested in non‐material‐based alternative business models too that provide the required benefit, service, or function. In practice, it begins with the question, “Do we need a product for a specific service and function, or is there a non‐material, more sustainable solution?” (Figure 4)—provided the function is actually needed and not merely desired. Knowledge‐based business models and leasing models are preferable to tonnage‐based ones. Sustainable chemistry and sustainable pharmacy take into account alternative behavior, that is, they favor intangible business models for generating revenue over the generation of tonnage. Some, such as chemical leasing, at least reduce the demand for material quantities. These are primarily function‐oriented business models, such as consultancy, training, and billing based on the number of items cleaned, the area disinfected, the surface area painted, or the service life of a catalyst, rather than on the quantity of cleaning agent, disinfectant, paint, or catalyst used, that is, sold [36, 184, 185, 186]. In other words, chemical‐based solutions are only the second choice if no convincing non‐material approach is available. The integration of chemistry and pharmacy into sustainability (“Sustainable Chemistry”, “Sustainable Pharmacy”) therefore begins with a broad, sustainability‐based holistic approach that takes into account the diverse interrelationships between sustainability and chemistry and pharmacy, respectively. On the one hand, they consider their material basis, including the associated processes as well as their products and their application, and, on the other hand, also social, ethical, and economic aspects.

**BOX 2: Key features of chemistry in the context of sustainability [183]**

1. Overall approach: Guide the chemical science and chemical industry to contribute to sustainability in accordance with sustainability principles, while understanding and taking into account broader contexts and potential interactions, including long‐range effects and temporal gaps between the chemical and other sectors.

2. Precautionary: Avoid, from the outset, the transfer of problems and costs to other sectors, spheres, and regions; prevent future contamination; and address past contamination, including the associated responsibilities.

3. Systemic thinking: Ensuring its interdisciplinary, multidisciplinary, and transdisciplinary nature, including a solid technical foundation, while taking other sectors into account to fully ensure sustainability. Application in industrial practice, including strategic and business planning, education, risk assessment, and other areas, including the social and economic sectors, across all stakeholder groups.

4. Ethical and social responsibility: Respect for the dignity of all inhabitants of the Earth, human rights, and the well‐being of all living beings; justice; and the interests of vulnerable groups; and the promotion of fair, inclusive, critical, and emancipatory approaches in all areas, including education, science, and technology.

5. Cooperation and transparency: Promoting exchange, cooperation, and the right to information for all stakeholders to improve the sustainability of business models, services, processes, and products, as well as related decisions, including environmental, social and economic development at all levels. Avoiding any form of “greenwashing” and “sustainability washing” through complete transparency in all scientific and business activities towards all stakeholders and civil society.

6. Sustainable and responsible innovation: A complete transformation of the chemical and related industries from the molecular to the macroscopic level of products, processes, functions, and services, adopting a proactive approach to sustainability, including continuous, trustworthy, transparent, and traceable monitoring.

7. Responsible management of chemicals: Promoting the responsible management of chemicals and waste throughout their entire life cycle, while avoiding toxicity, persistence, and bioaccumulation, as well as harm to humans and the environment caused by chemical substances, materials, processes, products, and services.

8. Circular economy: Taking into account the opportunities and limitations of a circular economy, including the reduction of overall substance, material, product, and associated energy flows across all spatial and temporal levels and dimensions, particularly with regard to scale and complexity.

9. Green chemistry: Adherence to as many of the 12 principles of green chemistry as possible within the framework of the sustainable application of chemistry, with a focus on hazard reduction whenever chemicals are required to provide a service or function, whenever and wherever this is compatible with sustainability.

10. Life cycle: Application of the above‐mentioned key characteristics to the entire life cycle of products, processes, functions, and services at all levels, for example, from the molecular to the macroscopic level and across all sectors, from a proactive perspective with regard to sustainability.

It would be unethical to apply different legal standards, such as continuing to manufacture a product that is banned in one country due to its toxicity, in order to export it to another country and sell it there [51, 187]. Further ethical questions include, for example: Who is affected by resource extraction or a chemical accident, whose environment is polluted, who pays for the clean‐up, and who receives the revenue from the use of the resource and the sale of the products: the local population or others? The same question could be posed regarding deforestation for the purpose of cultivating renewable raw materials or utilizing agricultural waste. Who is involved (and how) in which decisions? Who is affected by chemical waste and products end‐of‐life, and why are there different wages for the same professional activities? A sustainable approach considers that chemical synthesis takes place in a different location from raw material extraction or waste treatment and recycling, while also considering other issues relating to the end of a product's life, such as environmental pollution and all its consequences. Who has access to which product (e.g., medicines or pesticides), and who does not, and why not? Such questions are not simply brushed aside by a chemistry and pharmacy integrated with sustainability, nor are they referred to other sectors, the economy, or politics, nor is responsibility for them denied.

Sustainable chemistry and pharmacy recognize that the Earth is the foundation of everything, that the biosphere rests upon it, and that it in turn supports the anthroposphere—that part of the Earth system inhabited and utilized by humans—rather than everything being based on the economy. Without all this, our social sphere, upon which the technosphere and the economy rest, is not viable in the long term. Sustainable chemistry and pharmacy strive for the highest environmental, social, and ethical standards in all areas. The multi‐stakeholder approach and the global aspect of chemistry were recently addressed by UNEP in the Global Framework on Chemicals, including the Bonn Declaration [188]. How a systems approach can be applied at various levels has already been demonstrated [189, 190, 191, 192].

All of this must be applied by all stakeholders across the entire chemical and pharmaceutical sector, including downstream users, from services and resources through manufacturing and application of products, and right up to the end of their life cycle. Future professionals in the chemical and pharmaceutical industries and sciences must not only understand the underlying chemical and physical phenomena, but also possess knowledge from various scientific disciplines, particularly those relevant to sustainability, and be capable of interdisciplinary and transdisciplinary thinking and action. They must not view chemistry and pharmacy as science and practice solely from a material perspective [191]. Such thinking and understanding must also form part of future chemical and pharmaceutical education. Furthermore, it is essential to transform current educational models on all levels, as well as to provide further training for the workforce and to improve their knowledge and qualifications in a targeted manner, including the support and training of teachers in chemistry and related disciplines and subjects [193, 194, 195, 196, 197, 198, 199, 200, 201, 202].

## 7 Concrete Approach to Embed Chemistry and Pharmacy Into Sustainability

**FIGURE 5:**
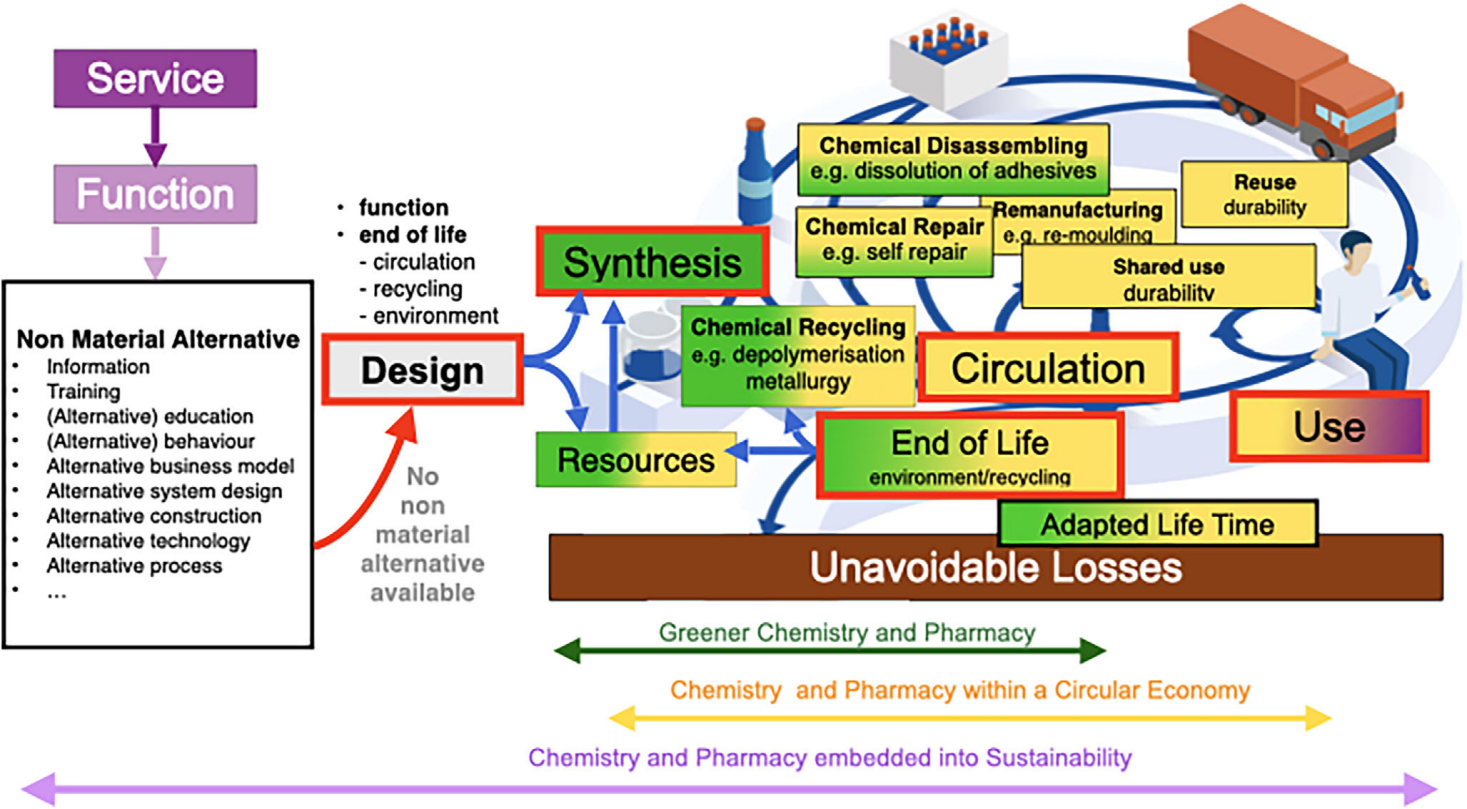
The areas of application for green, circular (circulation and recycling), and sustainable chemistry and pharmaceuticals (source of background image: European Parliament). They provide guidance on where sustainable chemical and pharmaceutical thinking, the circular economy, and circular chemistry and pharmacy, as well as green chemistry, play a role.

**FIGURE 6:**
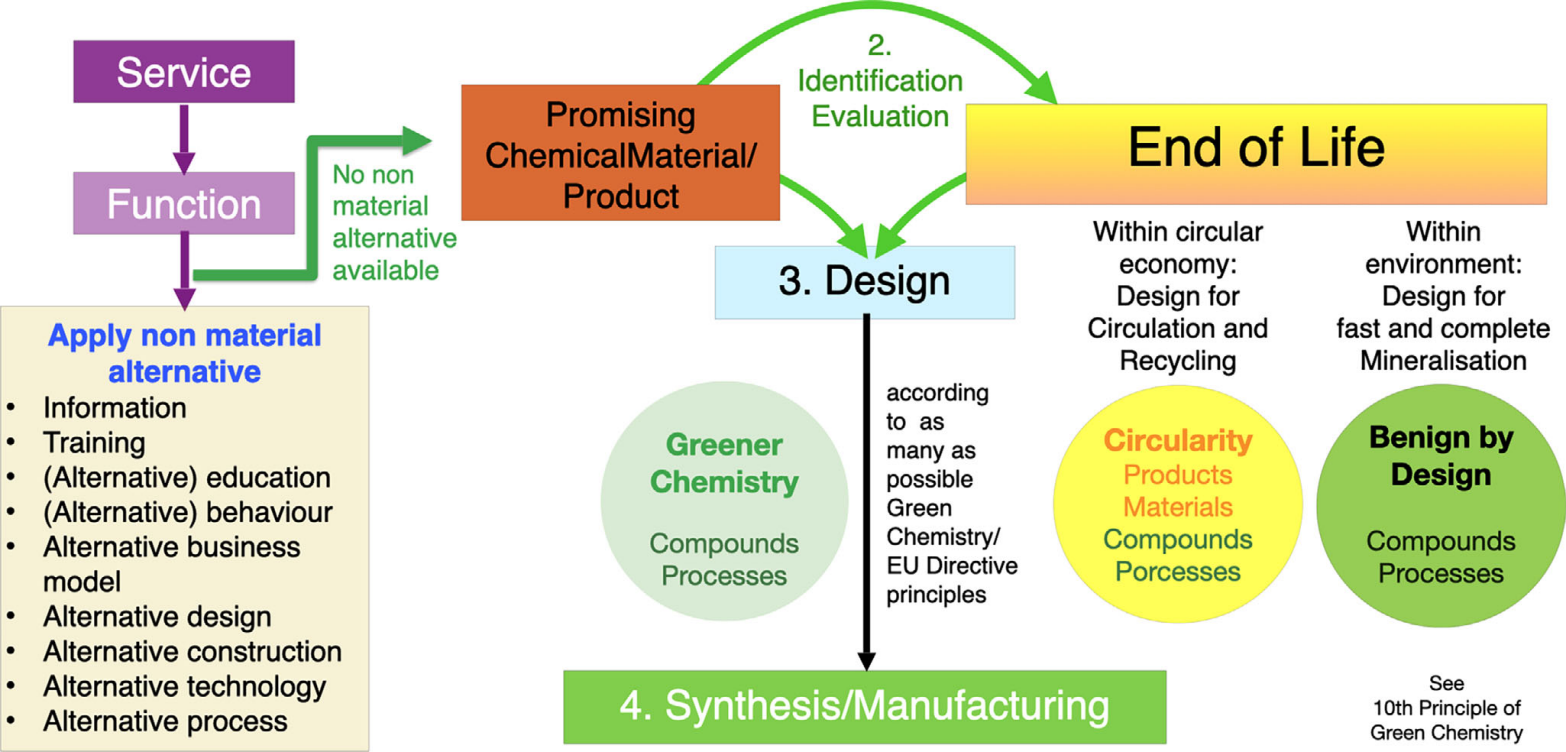
Detailed workflow for the application of chemistry and pharmacy within the context of sustainability to contribute to sustainability in a sustainable manner.

The ever‐increasing use of resources, the synthesis and manufacture of products, and the resulting generation of waste and environmental pollution stand in stark contrast to sustainability. As outlined above, sustainable pharmacy and sustainable chemistry form the overarching framework for pharmacy and chemistry to address this problem and steer practice towards a more sustainable chemistry and pharmacy. This recognizes that both green chemistry and pharmacy, as well as circular chemistry and pharmacy, are important tools for the synthesis and recycling of chemical and pharmaceutical products. Chemistry and pharmacy embedded in sustainability address their boundaries and areas of application but encompass far more (Figures 4, 5, 6). Figure 4 provides a general overview and a general workflow. Figure 5 offers a more detailed overview of the areas of application and potential contributions of green, circular, and sustainable pharmacy and chemistry, as well as their overlaps throughout the life cycle of a chemical or pharmaceutical service or product. Figure 6 outlines how the general approach shown in Figure 4 is put into practice.

The thought process and workflow always begin with the question of whether the desired service is actually needed. If so, is there a non‐material alternative that fulfills the identified service and function? If a material‐based service is required, it must be assessed right at the outset—that is, before synthesis—whether the product providing this service can be integrated into the circular economy and how. To this end, the principles of the circular economy and circular chemistry and pharmacy must be applied, and products must be designed in such a way that they remain in the cycle for as long as possible and can most easily be recycled later.

If pharmaceutical and chemical products cannot be kept within the cycle or returned to it or end up in the environment, they must be designed from the outset to degrade rapidly and completely there at the end of their life cycle (Principle No. 10 of Green Chemistry, “Benign by Design”), before being synthesized in accordance with the principles of Green Chemistry. Several tools are available for selecting more environmentally friendly chemicals as starting materials for synthesis [203, 204]. The question of how chemical products can be designed to be more sustainable has not yet been fully resolved [133, 134, 135, 136, 137]. An overview of potential indicators that are important for understanding what is more environmentally friendly, circular, and sustainable was recently published [205].

**BOX 3: The interplay of sustainable chemistry, circular chemistry, and green chemistry using the example of per‐ and polyfluoroalkyl substances (PFAS) [113]**

The group of PFAS in use comprises more than 10 000 different substances (https://echa.europa.eu/hot‐topics/perfluoroalkyl‐chemicals‐pfas). They are used because of the high stability of their C–F bond, as they remain stable even under extreme conditions, such as intense heat. They offer a wide range of properties, for example, as surfactants, water‐ and grease‐repellent agents and as modifiers in sealants that create hydrophobic surfaces and enable other properties. They are therefore used in many products, not least in the home. They are persistent, contaminate the environment, drinking water, and food, and accumulate in environmental organisms and humans. They are (eco)toxic. Their disposal is difficult and costly (see above). Their use must therefore be avoided wherever possible.

*Ski wax*

Service: reduced friction through hydrophobization, thereby increasing skiing speed; function: water‐repellency.

1. Avoiding ski wax containing PFAS, as well as completely avoiding ski wax altogether, affects all skiers equally (sport and leisure); higher friction resistance shifts the focus back to the athletes’ skills and fitness rather than on technology. Which is, of course, in keeping with the spirit of sport.
2. PFAS cannot be collected after use, that is, they can neither be returned to the cycle nor recycled. Circular chemistry does not work here.
3. If wax is desired (not to be confused with “required”), a wax should be used that is readily biodegradable in the environment (designed for this purpose) and synthesized according to the principles of green chemistry. It should fulfill as many of these principles as possible, not just one.

*Textile fibers*

Service: textiles stay dry longer and dry faster; Function: water‐repellency.

1. Such textiles are more likely to be needed in extreme working environments and perhaps for extreme outdoor activities, not in everyday life. Are these extreme activities necessary?
2. Designing textiles for a long service life and easy recovery; separate collection at the end of the service life to preserve and reuse these specific fibers.
3. Fibers can be made water‐repellent through a laser‐based nanostructuring treatment (non‐chemical alternative).
4. Use of natural waxes (considering where the wax resources come from, how they are extracted, and what impact their extraction has on biodiversity and local ecosystems [sustainable chemistry]) or synthetic polymers (synthesized according to as many principles of green chemistry as possible) to make them water‐repellent. It must be ensured that they can be easily separated during recycling (circular chemistry) and that they mineralize completely after being released into the environment (e.g., as microplastics) (Benign by Design).

*Hydrophobic surfaces of metal parts*

Service: water‐free surface, prevention of corrosion; Function: water‐repellency.

1. Metal surfaces can be made water‐repellent through laser‐abrasive treatment (non‐chemical alternative).
2. Such parts/products should be collected at the end of their service life for reuse or recycling.
3. Parts that cannot be made hydrophobic using a laser can be made water‐repellent by applying a polymer‐based surface coating. The design should allow for easy separation of the metal and polymer. Multiple materials should not be mixed to achieve hydrophobicity.
4. Environmentally friendly, biodegradable polymers should be used to account for abrasion and release into the environment. Synthesis of polymers in accordance with as many principles of green chemistry as possible. Consideration of where the resources for the polymers come from and how this affects the local environment, biodiversity, and community.

*Seals*

Service: sealing. Function: chemically inert, adaptable, temperature‐ and/or chemical‐resistant. Even though seals are only small parts, they can add up to thousands of tonnes (total material flow analysis: circular chemistry, sustainable chemistry).

1. What degree of sealing is required and what service life is necessary?
2. Is there a PFAS‐free seal (e.g., rubber, copper, brass) for the intended specific application?
3. Product design with regard to end‐of‐life collection and recycling, including easy separation of the seal from associated products. No mixing of multiple substances or materials to achieve the desired function. Use of recycled materials for new seals.
4. Where do the resources for the seals come from and what impact does this have on the local environment, biodiversity, and community?

Box 3 uses PFAS as an example to illustrate the interrelationships between green chemistry, circular chemistry, and sustainable chemistry. The starting point is always: Is the service actually needed? Is there a non‐material possibility of providing the service and fulfilling the function? PFAS, for example, are frequently used to make surfaces water‐repellent. In products such as ski wax or certain textiles, this service is not required. If necessary, a non‐material alternative may be available (see Box 3).

Another option, if required, would be a chemistry‐based approach that takes into account life‐cycle aspects such as circularity and recycling, as well as the fate of the chemical (e.g., ski wax synthesized in accordance with as many principles of green chemistry as possible, which mineralizes quickly and completely in the environment). For the hydrophobization of textile fibers or workpiece surfaces, hydrophobization using physical processes (e.g., microstructuring via laser treatment) is a suitable option. PFAS‐free packaging materials and, initially, assessing what packaging is required why for what purpose and for how long are alternatives in this area. PFAS‐free processes and membranes for the electrolysis of water to produce hydrogen are known. At least for some C‐CF_3_‐containing active pharmaceutical ingredients, it should be possible to replace these with C‐CF_3_‐free alternatives, e.g., fluoxetine with citalopram, paroxetine with sertraline, lansoprazole with omeprazole [205]. Their use avoids the release of trifluoroacetic acid (TFA) as a TP during degradation in the environment. TFA is a ubiquitous contaminant in food and all environmental compartments. It is highly persistent and toxic. Only with such an open‐minded approach to service provision, based on systems thinking, should products and their components be synthesised and manufactured in accordance with the 12 principles of green and circular chemistry, considering the limitations and potential misunderstandings outlined in Chapters 2–4.

## 8 Conclusions and Outlook

Greener chemistry and pharmacy, circular chemistry and pharmacy, and the complete embedding of pharmacy and chemistry into sustainability (“Sustainable Chemistry” and “Sustainable Pharmacy”) are neither synonyms nor interchangeable, respectively. Nor are they mutually exclusive. Greener chemistry and circular chemistry are indispensable components of sustainable chemistry and pharmacy, but the overarching framework is sustainable chemistry and pharmacy. The latter are interdisciplinary and embedded in sustainability. The starting point must be a holistic systems approach and understanding that encompasses the entire life cycle of products and their impact on sustainability, starting with service and function and beginning with non‐material alternatives. A mindset that focuses exclusively on value chains is far too short‐sighted, as value chains often end where there is no longer any economic value, meaning they often do not take waste or social aspects into account.

Until now, the chemical and pharmaceutical industries have mostly strived to produce ever newer and often more complex molecules, materials, and products. The economization of science, measured by impact factors, only adds to this. This trend continues in the industries that use the products of the chemical and pharmaceutical sectors for their own products. In recent decades, the number of products on the market has increased enormously in terms of both quantity and variety, including product types and the number of components. They are often advertised as significant improvements or even as entirely new products. These products often increased performance by only a few percent while causing a disproportionately high rise in material and energy consumption and waste generation. These unsustainable practices must be abandoned.

A simpler product will usually be cheaper, and if a cheaper product can achieve the same result (or at least come close to what is actually needed), it will generally outperform its more expensive competitors. There are also examples where a product contains fewer chemicals and is even sold at a higher price because customers are demanding more environmentally friendly and sustainable products and are willing to pay for them, such as silicone‐free hair conditioners, biocide‐free exterior paints, and food without artificial flavorings. These examples show that there is no general contradiction between more sustainable chemistry, pharmaceuticacy and revenue, not to mention economic costs: from a systemic perspective that takes all external costs into account, greater sustainability is always beneficial.

It is high time to fully embed today's chemical and pharmaceutical industries within sustainability, starting with the question of whether a service or function is required, to understand the commonalities, overlaps, and differences between green, circular, and sustainable chemistry and pharmacy, and to help them fulfill their promises.

## Author Contributions

**Klaus Kümmerer**: conceptualization, data curation, formal analysis, visualization, writing – original draft, methodology, investigation, writing – review and editing, funding acquisition, validation, project administration, resources.

## Conflicts of Interest

The author declares no conflicts of interest.

## Data Availability

The data that support the findings of this study are available on request from the corresponding author. The data are not publicly available due to privacy or ethical restrictions.

## References

[1] United Nations Environment Programme , Global Chemicals Outlook II—From Legacies to Innovative Solutions: Implementing the 2030 Agenda for Sustainable Development, (United Nations Environment Programme, 2019), accessed September 23, https://www.unep.org/topics/chemicals‐and‐pollution‐action/chemicals‐management/global‐chemicals‐outlook.

[2] GIZ , Deutsche Gesellschaft für Internationale Zusammenarbeit GmbH, GIZ Fact Sheet Sustainable Chemistry for Climate Protection, (GIZ, 2023), accessed September 23), https://www.isc3.org/cms/wp‐content/uploads/2023/11/Factsheet_ClimateProtection_181023.pdf.

[3] S. A. Matlin , G. Mehta , S. E. Cornell , A. Krief , and H. Hopf , “Chemistry and Pathways to Net Zero for Sustainability,” RSC Sustainability 1 (2023): 1704–1721.

[4] Science Based Targets Initiative (SBTi) , Chemicals Sector, (SBTi, 2025), accessed September 23, https://sciencebasedtargets.org/sectors/chemicals.

[5] F. Krausmann , S. Gingrich , N. Eisenmenger , K.‐H. Erb , H. Haberl , and M. Fischer‐Kowalski , “Growth in Global Materials Use, GDP and Population During the 20th Century,” Ecological Economics 68 (2009): 2696–2705.

[6] W. Steffen , W. Broadgate , L. Deutsch , O. Gaffney , and C. Ludwig , “The Trajectory of the Anthropocene: The Great Acceleration,” Anthropocene Review 2 (2015): 81–98.

[7] E. Elhachami , L. Ben‐Uri , J. Grozovski , Y. M. Bar‐On , and R. Milo , “Global Human‐Made Mass Exceeds All Living Biomass,” Nature 588 (2020): 442–444. 33299177 10.1038/s41586-020-3010-5

[8] V. Zepf , A. Reller , C. Rennie , M. Ashfield , and J. Simmons , Materials Critical to the Energy Industry. An Introduction, 2nd ed. (BP plc, 2014).

[9] J. G. Martínez , “Why We Need a Common Vision for Chemitry,” Chemical & Engineering News 98 (2020): 22.

[10] Z. Wang , G. W. Walker , D. C. G. Muir , and K. Nagatani‐Yoshida , “Toward a Global Understanding of Chemical Pollution: A First Comprehensive Analysis of National and Regional Chemical Inventories,” Environmental Science & Technology 54 (2020): 2575–2584. 31968937 10.1021/acs.est.9b06379

[11] E. L. Schymanski , J. Zhang , P. A. Thiessen , P. Chirsir , T. Kondic , and E. E. Bolton , “Per‐ and Polyfluoroalkyl Substances (PFAS) in PubChem: 7 Million and Growing,” Environmental Science & Technology 57 (2023): 16918–16928. 37871188 10.1021/acs.est.3c04855 PMC10634333

[12] European Chemicals Agency (ECHA) , Per‐ and Polyfluoroalkyl Substances (PFAS), (ECHA, 2025), accessed February 25, https://echa.europa.eu/hot‐topics/perfluoroalkyl‐chemicals‐pfas.

[13] H. Wiesinger , Z. Wang , and S. Hellweg , “Deep Dive Into Plastic Monomers, Additives, and Processing Aids,” Environmental Science & Technology 55 (2021): 9339–9351. 34154322 10.1021/acs.est.1c00976

[14] UNEP , United Nations Environment Programme and Secretariat of the Basel, Rotterdam and Stockholm Conventions. Chemicals in Plastics: A Technical Report (UNEP, 2023), accessed September 25, https://www.unep.org/resources/report/chemicals‐plastics‐technical‐report.

[15] F. Sommer , V. Dietze , A. Baum , et al., “Tire Abrasion as a Major Source of Microplastics in the Environment,” Aerosol and Air Quality Research 18 (2018): 2014–2028.

[16] D. Stradling and R. Stradling , “Perceptions of the Burning River: Deindustrialisation and Cleveland's Cuyahoga River,” Environmental History 13 (2008): 515–535.

[17] University of Buffalo , Love Canal: Timeline and Photos (University of Buffalo, 2021), https://research.lib.buffalo.edu/love‐canal/timeline‐and‐photos.

[18] R. Carson , Silent Spring (Houghton Mifflin Company, 1962).

[19] J. Moore and E. A. Moore , Environmental Chemistry (Academic Press, 1976).

[20] M. J. Molina and F. S. Rowland , “Stratospheric Sink for Chlorofluoromethanes: Chlorine Atom‐Catalysed Destruction of Ozone,” Nature 249 (1974): 810–812.

[21] United Nations Environment Programme , The Montreal Protocol (UNEP, 2020), https://www.unep.org/ozonaction/who‐we‐are/about‐montreal‐protocol.

[22] Stockholm Convention Secretariat (United Nations Environment Programme) , Stockholm Convention on Persistent Organic Pollutants (POPs) – Overview, (UNEP, 2020), https://www.pops.int/TheConvention/Overview/tabid/3351/Default.aspx.

[23] G. Goldenman , M. Fernandes , M. Holland , et al., The Cost of Inaction. A Socioeconomic Analysis of Environmental and Health Impacts Linked to Exposure to PFAS (Nordic Council of Ministers/Publication Unit, 2019), https://norden.diva‐portal.org/smash/get/diva2:1295959/FULLTEXT01.pdf.

[24] V. Obsekov , L. G. Kahn , and L. Trasande , “Leveraging Systematic Reviews to Explore Disease Burden and Costs of per‐ and Polyfluoroalkyl Substance Exposures in the United States,” Exposure and Health 15 (2023): 373–394. 37213870 10.1007/s12403-022-00496-y PMC10198842

[25] POLITICO , “Forever Chemicals” Are Everywhere. The Battle Over Who Pays to Clean Them Up Is Just Getting Started (POLITICO, 2025), accessed February 13, https://www.politico.com/news/2022/09/13/the‐battle‐over‐who‐pays‐to‐clean‐up‐chemicals‐00056136.

[26] T. Perkins , Societal Cost of “Forever Chemicals” About $17.5tn Across Global Economy—Report (Guardian, 2023), accessed February 13, Proceedings of an International Seminar Organized by the Senior Advisers to ECE Governments on Environmental Problems on the Principles and Creation of Non‐Waste Technology and Production, Paris, 29 November–4 December 1, https://amp.theguardian.com/environment/2023/may/12/pfas‐forever‐chemicals‐societal‐cost‐new‐report.

[27] S. Finckh , E. Carmona , D. Borchardt , et al., “Mapping Chemical Footprints of Organic Micropollutants in European Streams,” Environment International 183 (2024): 108371. 38103345 10.1016/j.envint.2023.108371

[28] World Health Organization Regional Office for Europe , Human Biomonitoring: Facts and Figures (WHO Regional Office for Europe, 2015), https://iris.who.int/bitstream/handle/10665/164588/WHO‐EURO‐2015‐3209‐42967‐60040‐eng.pdf?sequence=3.

[29] A. C. Johnson , X. Jin , N. Nakada , and J. P. Sumpter , “Learning From the Past and Considering the Future of Chemicals in the Environment,” Science 367 (2020): 384–387. 31974243 10.1126/science.aay6637

[30] E. Kristiansson , J. Coria , L. Gunnarsson , and M. Gustavsson , “Does the Scientific Knowledge Reflect the Chemical Diversity of Environmental Pollution?—A Twenty‐Year Perspective,” Environmental Science & Policy 126 (2021): 90–98.

[31] P. J. Landrigan , R. Fuller , N. J. R. Acosta , et al., “The Lancet Commission on Pollution and Health,” Lancet 391 (2018): 462–512. 29056410 10.1016/S0140-6736(17)32345-0

[32] B. Geueke , L. V. Parkinson , K. J. Groh , et al., “Evidence for Widespread Human Exposure to Food Contact Chemicals,” Journal of Exposure Science & Environmental Epidemiology 35 (2025): 330–341. 39285208 10.1038/s41370-024-00718-2 PMC12069106

[33] K. Kümmerer , ed., Pharmaceuticals in the Environment, 3rd ed. (Springer, 2008).

[34] J. L. Wilkinson , A. B. A. Boxall , D. Kolpin , et al., “Pharmaceutical Pollution of the World's Rivers,” Proceedings of the National Academy of Sciences of the United States of America 119 (2022): e2113947119. 35165193 10.1073/pnas.2113947119 PMC8872717

[35] K. Kümmerer , Green and Sustainable Pharmacy, edited by M. Hempel (Springer, 2010).

[36] K. Witte and M. Müller , “Sustainable Pharmacy‒a Guiding Principle,” Sustainable Chemistry and Pharmacy 44 (2025): 101897.

[37] United Nations Economic Commission for Europe , Non‐Waste Technology and Production. A Seminar of the United Nations Economic Commission for Europe. Pergamon (Pergamon Press, 1978).

[38] T. Kletz , “What You Don't Have Can't Leak,” Chemistry and Industry (1978): 287–291.

[39] M. G. Royston , Pollution Prevention Pays (Pergamon, 1979).

[40] M. A. Murphy , “Early Industrial Roots of Green Chemistry and the History of the BHC Ibuprofen Process Invention and Its Quality Connection,” Found Chemistry 20 (2018): 121–165.

[41] M. A. Murphy , “Early Industrial Roots of Green Chemistry—II,” Substantia 4 (2020): 15–57.

[42] M. A. Murphy , “Early Industrial Roots of Green Chemistry,” Chemistry International 43 (2021): 21–25.

[43] L. Maxim , “The Birth of Green Chemistry: A Political History,” Science, Technology & Human Values 50 (2023): 144–168.

[44] M. Krasnodębski , “Reinventing the Wheel: A Critical Look at One‐World and Circular Chemistries,” Studies in History and Philosophy of Science 96 (2022): 112–120. 36206586 10.1016/j.shpsa.2022.09.004

[45] M. Krasnodębski , “Lost Green Chemistries: History of Forgotten Environmental Trajectories,” Centaurus 64 (2022): 509–536.

[46] W. Rabsch and W. Fritsche , “Physiologie des Inilinabbaus durch Achrombacter Ir 2,,” Zeitschrift für Allgemeine Mikrobiologie 17 (1977): 139–148. 868083 10.1002/jobm.3630170207

[47] J. H. Clark , A. P. Kybett , D. J. Macquarrie , S. J. Barlow , and P. Landon , “Montmorillonite Supported Transition Metal Salts as Friedel–Crafts Alkylation Catalysts,” Chemical Communications 18 (1989): 1353–1354.

[48] F. Trotta , P. Tundo , and G. Moraglio , “Selective Mono‐N‐alkylation of Aromatic Amines by Dialkyl Carbonate Under Gas‐liquid Phase‐Transfer Catalysis (GL‐PTC) Conditions,” The Journal of Organic Chemistry 52 (1987): 1300–1304.

[49] P. Tundo and F. Aricó , “Green Chemitry on the Rise: Thoughts on the Short History of the Field,” Chemistry International 29 (2007): 4–7.

[50] R. A. Sheldon , Chemistry and Industry (1992): 903–906.

[51] World Commission on Environment and Development , Our Common Future (Brundtland Report), http://un‐documents.net/our‐common‐future.pdf.

[52] United States of America Environmental Protection Agency , Basics of Green Chemistry (United States of America Environmental Protection Agency, 2025), https://www.epa.gov/greenchemistry/basics‐green‐chemistry.

[53] United States Environmental Protection Agency , Pollution Prevention Act of 1990 (United States Environmental Protection Agency, 2025), https://www.epa.gov/p2/pollution‐prevention‐act‐1990.

[54] United Nations , Report of the United Nations Conference on Environment and Development 1989, Volume 1 Resolutions Adopted by the Conference (United Nations, 2025), https://docs.un.org/en/A/CONF.151/26/Rev.1(vol.I).

[55] Organisation for Economic Co‐operation and Development , Proceedings of the OECD workshop on sustainable chemistry (OECD, 1998).

[56] J. A. Linthorst , “An Overview: Origins and Development of Green Chemistry,” Foundations of Chemistry 12 (2010): 55–68.

[57] D. Lenoir , K.‐W. Schramm , and J. O. Lalah , “Green Chemistry: Some Important Forerunners and Current Issues,” Sustainable Chemistry and Pharmacy 18 (2020): 100313.

[58] M. Krasnodębski , “An Unlikely Bifurcation: History of Sustainable (But Not Green) Chemistry,” Foundations of Chemistry 25 (2023): 463–484.

[59] I. Pasquon and L. Zanderighi , La Chimica verde (Hoepli, 1987).

[60] D. Mackenzie , New Scientist , November 25 (1989).

[61] Council of the European Union , Council Directive 96/61/EC of 24 September 1996 Concerning Integrated Pollution Prevention and Control (Council of the European Union, 1996), https://eur‐lex.europa.eu/legal‐content/EN/TXT/?uri=CELEX%3A31996L0061.

[62] P. T. Anastas and J. C. Warner , Green Chemistry Theory and Practice (Oxford University Press, 1998).

[63] J. Robinson , “In situ with Paul Anastas,” Chemistry World 21 (2024): 61.

[64] J. P. Dinh , “Scientist You Should Know: Paul Anastas Is the Father of Green Chemistry,” Discover Magazine,13 July 2022), https://www.discovermagazine.com/the‐sciences/scientist‐you‐should‐know‐paul‐anastas‐is‐the‐father‐of‐green‐chemistry.

[65] Volvo , Paul Anastas wins the Volvo Environment Prize 2021 (Volvo, 2021), https://www.environment‐prize.com/laureates/paul‐anastas/.

[66] T. J. Collins , “Green Chemistry,” in Encyclopaedia of Chemistry, edited by J. J. Lagowski , (Simon and Schuster, 1997), 691–697.

[67] A. von Gleich , Der wissenschaftliche Umgang mit der Natur. Über die Vilefalt harter und sanfter Naturwissenschaften (Campus Verlag, 1989).

[68] H. Fischer , Ein Plädoyer für eine sanfte Chemie. Über den nachhaltigen Gebrauch der Stoffe (Verlag C. F. Müller, 1993).

[69] O. Hutzinger , “The Greening of Chemistry — Is It Sustainable?” Environmental Science and Pollution Research 6 (1999): 123. 19009378 10.1007/BF02987605

[70] H. Krähling , “Green vs. sustainable Chemistry — More Than a Discussion on Catchwords,” Environmental Science and Pollution Research 6 (1999): 124. 19009379 10.1007/BF02987606

[71] C. A. Marques and A. A. S. C. Machado , “Environmental Sustainability: Implications and Limitations to Green Chemistry,” Found Chemistry 16 (2024): 125–147.

[72] T. E. Graedel , “Green Chemistry and Sustainable Development,” in Handbook of Green Chemistry and Technology, edited by J. H. Clark and D. Macquarrie (Wiley‐Blackwell, 2002), 56–61.

[73] A. S. De Souza , P. G. Ferreira , I. S. de Jesusa , A. S. de Carvalhoa , D. O. Futuro , and V. F. Ferreiraa , Quimica Nova 47 (2024): e20240049.

[74] J. Roberts , “Creating Green Chemistry: Discursive Strategies of a Scientific Movement” (PhD thesis, Faculty of Virginia Polytechnic Institute and State University, 2006).

[75] M. Krasnodębski , “The Bumpy Road to Sustainability: Reassessing the History of the Twelve Principles of Green Chemistry,” Studies in History and Philosophy of Science 103 (2024): 85–94. 38091644 10.1016/j.shpsa.2023.12.001

[76] B. M. Trost , “The Atom Economy—A Search for Synthetic Efficiency,” Science 254 (1991): 1471–1477. 1962206 10.1126/science.1962206

[77] R. A. Sheldon , “The E Factor 25 Years On: The Rise of Green Chemistry and Sustainability,” Green Chemistry 19 (2017): 18–43.

[78] J. H. Clark , “Green Chemistry Metrics — What, Why and Using Them Today,” Nature Reviews Methods Primers 5 (2025): 63.

[79] K. Kümmerer and M. Held , “Die Umweltwissenschaften im Kontext von Zeit‐Begriffe unter dem Aspekt der Zeit,” UWSF‐Zeitschrift für Umweltchemie und Ökotoxikologie 9 (1997): 169–178.

[80] J. Huo , Z. Wang , P. Lauri , J. D. Medrano‐García , G. Guillén‐Gosálbez , and S. Hellweg , “Region‐Specific Sourcing of Lignocellulose Residues as Renewable Feedstocks for a Net‐Zero Chemical Industry,” Environmental Science & Technology 58 (2024): 13748–13759. 39049709 10.1021/acs.est.4c03005 PMC11308523

[81] D. Pleissner and K. Kümmerer , “Green Chemistry and Its Contribution to Industrial Biotechnology,” in Sustainability and Life Cycle Assessment in Industrial Biotechnology. Advances in Biochemical Engineering/Biotechnology, edited by M. Fröhling and M. Hiete (Springer, 2018), 281–298. 10.1007/10_2018_73 30270411

[82] S. Baumberger and M.‐C. Scherrmann , Green Chemistry, Eco‐Friendly Chemistry, Biorefinery. In Green Chemistry and Agro‐food Industry: Towards a Sustainable Bioeconomy, edited by S. Baumberger (Springer Cham, 2024), 3–22.

[83] R. A. Sheldon , “Catalysis: The Key to Waste Minimization,” Journal of Chemical Technology & Biotechnology 68 (1997): 381–388.

[84] K. Kümmerer , D. D. Dionysiou , O. Olsson , and D. Fatta‐Kassinos , Science of the Total Environment 652 (2018): 836–850. 30380490 10.1016/j.scitotenv.2018.10.219

[85] J. E. Lovelock , “Atmospheric Fluorine Compounds as Indicators of Air Movements,” Nature 230 (1971): 379–379.

[86] J. Makkor , M. Palm , I. Pardo Cantos , et al., “First Measurements of CFC‐12 in 1951 at Jungfraujoch and Comparison to Current Measurements and Atmospheric Models,” Geophysical Research Letters 53 (2026): e2025GL117453.

[87] Wissenschaftlicher Dienst des Deutschen Bundestags , 2009.

[88] ACS Green Chemistry Institute , ACS GCI Pharmaceutical Roundtable (ACS Green Chemistry Institute, 2025), https://acsgcipr.org/.

[89] K. Polanyi , The Great Transformation (Rinehart & Company Inc., 1944).

[90] F. Soddy , Virtual Wealth and Debt: The Solution of the Economic Paradox (Omnia Veritas Ltd, 2021). Original work published 1926.

[91] N. Georgescu‐Roegen , The Entropy Law and the Economic Process (Harvard University Press, 1971).

[92] H. E. Daly , Toward a Steady‐state Economy (W. H. Freeman, 1973).

[93] M. Faber , “How to be an Ecological Economist,” Ecological Economics 66 (2008): 1–7.

[94] B. Commoner , Closing the Circle: Nature, Man, and Technology (Alfred A. Knopf, 1971).

[95] W. R. Stahel , “The Product‐life factor,” in An Inquiry into the Nature of Sustainable Societies: The Role of the Private Sector, edited by S. Grinton (Houston Area Research Center, 1982), 72–104.

[96] W. R. Stahel , “,The Ciruclar Economy,” Nature 531 (2016): 435–438. 27008952 10.1038/531435a

[97] Ellen MacArthur Foundation , Timeline (Ellen MacArthur Foundation, 2025), https://www.ellenmacarthurfoundation.org/about‐us/timeline.

[98] W. McDonough and M. Braungart , Cradle to Cradle: Remaking the Way We Make Things (North Point Press, 2002).

[99] M. Braungart , W. McDonough , and A. Bollinger , “Cradle‐to‐Cradle Design: Creating Healthy Emissions—A Strategy for Eco‐Effective Product and System Design,” Journal of Cleaner Production 15 (2007): 1337–1348.

[100] Y. Geng , J. Sarkis , and R. Bleischwitz , “How to Build a Circular Economy for Rare‐Earth Elements,” Nature 619 (2023): 248–252. 37430110 10.1038/d41586-023-02153-z

[101] T. Watari , K. Nansai , and K. Nakajima , “Review of Critical Metal Dynamics to 2050 for 48 Elements,” Resources, Conservation and Recycling 155 (2020): 104669.

[102] International Energy Agency , “The Role of Critical Minerals in Clean Energy Transitions” (2022), https://www.iea.org/reports/the‐role‐of‐critical‐minerals‐in‐clean‐energy‐transitions.

[103] A. Stubbins , K. Lavender , S. E. Munoz , T. S. Bianchi , and L. Zhu , “Plastics n the Earth System,” Science 377 (2021): 351–355. 10.1126/science.abb0354 34210876

[104] J. Mangers , M. Minoufekr , P. Plapper , and S. Kolla , “An Innovative Strategy Allowing a Holistic System Change towards Circular Economy Within Supply‐Chains,” Energies 14 (2021): 4375.

[105] J. Korhonen , A. Honkasalo , and J. Seppälä , “Circular Economy: The Concept and Its Limitations,” Ecological Economics 143 (2018): 37–46.

[106] United Nations Environment Programme , United Nations Environment Programme Green and Sustainable Chemistry Framework Manual (United Nations Environment Programme, 2019), https://www.unep.org/resources/toolkits‐manuals‐and‐guides/green‐and‐sustainable‐chemistry‐framework‐manual.

[107] T. Zink and R. Geyer , “Circular Economy Rebound,” Journal of Industrial Ecology 21 (2017): 593–602.

[108] M. Linder , “Ripe for Disruption: Reimagining the Role of Green Chemistry in a Circular Economy,” Green Chemistry Letters and Reviews 10 (2017): 428–435.

[109] T. Keijer , V. Bakker , and J. C. Slootweg , “Circular Chemistry to Enable a Circular Economy,” Nature Chemistry 11 (2019): 190–195. 10.1038/s41557-019-0226-9 30792512

[110] A. Lansink , Challenging Changes—Connecting Waste Hierarchy and Circular Economy (LEA Nijmegen, 2017).

[111] A. Lansink , “Challenging Changes—Connecting Waste Hierarchy and Circular Economy,” Waste Management & Research 36 (2018): 872.

[112] J. Wesselkämper , T. P. Hendrickson , S. Lux , and S. von Delft , “Recycling or Second Use? Supply Potentials and Climate Effects of End‐of‐Life Electric Vehicle Batteries,” Environmental Science & Technology 59 (2025): 15751–15765. 40700652 10.1021/acs.est.5c01823 PMC12329728

[113] K. Kümmerer , “Orienting Chemistry Towards Sustainability,” in Chemistry Education for a Sustainable Future, edited by C. Middlecamp , M. Kirchhoff , P. Mahaffy , and K. Kümmerer (Royal Society of Chemistry, 2025).

[114] J. H. Clark , T. J. Farmer , L. Herrero‐Davila , and J. Sherwood , “Circular Economy Design Considerations for Research and Process Development in the Chemical Sciences,” Green Chemistry 18 (2017): 3914–3934.

[115] K. Kümmerer , J. H. Clark , and V. G. Zuin , “Rethinking Chemistry for a Circular Economy,” Science 367 (2020): 369–370. 31974237 10.1126/science.aba4979

[116] R. W. Moore , Physical Chemistry, 4th ed. (Prentice Hall Inc., 1972).

[117] G. Nicolis and I. Prigogine , Self‐Organisation in Non‐Equilibrium Systems: From Dissipative Structures to Order Through Fluctuations (Wiley, 1977).

[118] R. Schoemaker , C. Sun , D. Chiarugi , T. Tyrikos‐Ergas , and P. H. Seeberger , “Chemistries Moonshot: An Entirely Recyclable Car,” ACS Central Science 11 (2025): 1052–1061. 40726800 10.1021/acscentsci.5c00589 PMC12291114

[119] G. Giampietro , “The Entropic Nature of the Economic Process: A Scientific Explanation of the Blunder of the Circular Economy,” in The Impossibilities of the Circular Economy, edited by H. Lehmann , C. Hinske , V. de Margerie , and A. S. Nikolova (Routledge, 2023), 37–47.

[120] R. De Man , “Circularity Dreams: Denying Physical realities,” in The Impossibilities of the Circular Economy, edited by H. Lehmann , C. Hinske , V. de Margerie , and A. Slaveikovka Nikolova (Routledge, 2023), 3–10.

[121] T. Zimmermann and S. Gössling‐Reisemann , “Critical Materials and Dissipative Losses: A Screening Study,” Science of the Total Environment 461 (2013): 774–780. 23768419 10.1016/j.scitotenv.2013.05.040

[122] H. Friege and K. Kümmerer , “Practising Circular Economy: Stumbling Blocks for Circulation and Recycling,” in The Impossibilities of the Circular Economy, edited by H. Lehmann , C. Hinske , V. de Margerie , and A. Slaveikovka Nikolova (Routledge, 2023), 259–271.

[123] J. Huether , C. Joachimsthaler , and M. Faulstich , “The Impossibility of Circular Recycling,” in The Impossibilities of the Circular Economy, edited by H. Lehmann , C. Hinske , V. de Margerie , and A. Slaveikovka Nikolova (Routledge, 2023), 48–58.

[124] M. Held and A. Reller , “The Material Prerequisites of the Energy Transition in the Great Transformation,” in Critical Metals in the Great Transformation, edited by H. Exner , M. Held , and K. Kümmerer (Springer Spektrum, 2016), 109–137.

[125] K. Kümmerer , “Concentration, Functionality and Dissipation—Basic Categories for Understanding the Availability of Metallic Raw Materials,” in Critical Metals in the Great Transformation, edited by H. Exner , M. Held , and K. Kümmerer (Springer Spektrum, 2016), 53–86.

[126] V. M. Sanz , A. M. Serrano , and M. Schlummer , “A Mini‐Review of the Physical Recycling Methods for Plastic Parts in End‐of‐Life Vehicles,” Waste Management & Research 40 (2022): 1757–1765. 35708148 10.1177/0734242X221094917

[127] I. Vollmer , M. J. F. Jenks , M. C. P. Roelands , et al., “Beyond Mechanical Recycling: Giving New Life to Plastic Waste,” Angewandte Chemie International Edition 59 (2020): 15402–15423. 32160372 10.1002/anie.201915651 PMC7497176

[128] H. Grassl , “Environmental and Climate Research,” in Ecology of Time: On Finding the Right Time Scales, edited by M. Held , K. A. Geißler and S. Hirzel (Wissenschaftliche Verlagsgesellschaft, 1993), 75–84.

[129] K. Kümmerer , “Times of Nature—Times of Man,” in Ecology of Time: On Finding the Right Measures of Time, edited by M. Held , K. A. Geißler , and S. Hirzel (Wissenschaftliche Verlagsgesellschaft, 1993), 85–104.

[130] K. Kümmerer , “The Ecological Impact of Time,” Time & Society 5 (1996): 209–235.

[131] B. Adam , Timescapes of Modernity. The Environment and Invisible Hazards (Routledge, 1998).

[132] W. Stahel , “The Strategy of Durability,” in Ecology of Time: On Finding the Right Measure of Time, edited by M. Held , K. A. Geißler , and S. Hirzel (Wissenschaftliche Verlagsgesellschaft, 1993), 105–110.

[133] P.‐G. Rieger , H. M. Meier , M. Gerle , U. Vogt , T. Groth , and H. J. Knackmuss , “Xenobiotics in the Environment: Present and Future Strategies to Obviate the Problem of Biological Persistence,” Journal of Biotechnology 94 (2002): 101–123. 11792455 10.1016/s0168-1656(01)00422-9

[134] R. S. Boethling , “Designing Safer Chemicals. Green Chemistry for Pollution Prevention,” in Designing Biodegradable Chemicals, edited by C. S. DeVito and R. L. Garrett (American Chemical Society, 1996), 156–171, ACS Symposium Series.

[135] K. Kümmerer , “Sustainable from the Very Beginning: Rational Design of Molecules by Life Cycle Engineering as an Important Approach for Green Pharmacy and Green Chemistry,” Green Chemistry 9 (2007): 899–907.

[136] C. Leder , M. Suk , S. Lorentz , et al., “Reducing Environmental Pollution by Antibiotics Through Design for Environmental Degradation,” ACS Sustainable Chemistry & Engineering 9 (2021): 9358–9368.

[137] M. Suk and K. Kümmerer , “Towards Greener and Sustainable Ionic Liquids Using Naturally Occurring and Nature‐Inspired Pyridinium Structures,” Green Chemistry 25 (2023): 365–374.

[138] C. G. Daughton , “Cradle‐to‐cradle Stewardship of Drugs for Minimizing Their Environmental Disposition While Promoting human Health. I. Rationale for and Avenues Toward a Green Pharmacy,” Environ Health Perspectives 111 (2002): 757–774. 10.1289/ehp.5947 PMC1241487 12727606

[139] United Nations Environment Programme , Sustainable Development Goals (SDGs) (United Nations Environment Programme, 2015), https://sdgs.un.org/goals.

[140] J. Rockström , J. W. Steffen , K. Noone , et al., “Planetary Boundaries: Exploring the Safe Operating Space for Humanity,” Ecology and Society 14 (2009): 32.

[141] J. Rockström , J. Gupta , D. Quin , et al., “Safe and Just Earth System Boundaries,” Nature 619 (2023): 102–111. 37258676 10.1038/s41586-023-06083-8 PMC10322705

[142] J. Huber , “Towards Industrial Ecology: Sustainable Development as a Concept of Ecological Modernization,” Journal of Environmental Policy & Planning 2 (2000): 269–285.

[143] M. Rudolf and M. Schmidt , “Efficiency, Sufficiency and Consistency in Sustainable Development: Reassessing Strategies for Reaching Overarching Goals,” Ecological Economics 227 (2025): 108426.

[144] K. G. Steinhäuser and M. G. Ophoff , “The Need for Change: A Roadmap for the Sustainable Transformation of the Chemical Industry,” Sustainable Chemistry 6 (2025): 16.

[145] L. Mederake , “Without a Debate on Sufficiency, a Circular Plastics Economy Will Remain an Illusion,” Circular Economy and Sustainability 3 (2023): 1425–1439. 10.1007/s43615-022-00240-3 PMC9749638 36531658

[146] H. Jonas , The Imperative of Responsibility: In Search of an Ethics for the Technological Age (University of Chicago Press, 1984).

[147] J. Kovac , The Ethical Chemist: Professionalism and Ethics in Science, 2nd ed. (Oxford University Press, 2018).

[148] H. Frank , L. Campanella , F. Dondi , et al., “Ethics, Chemistry, and Education for Sustainability,” Angewandte Chemie International Edition 50 (2022): 8482–8490. 10.1002/anie.201007599 21796744

[149] J. Mehlich , F. Moder , B. Van Tiggelen , L. Campanella , and H. Hopf , “The Ethical and Social Dimensions of Chemistry: Reflections, Considerations, and Clarifications,” Chemistry – A European Journal 23 (2017): 1210–1218. 28105743 10.1002/chem.201605259

[150] Ethics in Chemistry , EUChemS Working Party on Ethics in Chemistry, https://www.euchems.eu/divisions/ethics‐in‐chemistry/.

[151] S. Matlin , G. Mehta , H. Hopf , and A. Grief , “One‐world Chemistry and Systems Thinking,” Nature Chemistry 8 (2016): 393–398. 10.1038/nchem.2498 27102668

[152] D. J. C. Constable , “Green and Sustainable Chemistry—The Case for a Systems‐Based, Interdisciplinary Approach,” iScience 24 (2021): 103489. 34934914 10.1016/j.isci.2021.103489 PMC8654969

[153] United Nations , Report of the World Summit on Sustainable Development (United Nations, 2002).

[154] United Nations Environment Programme , Global Framework on Chemicals, https://www.chemicalsframework.org/.

[155] World Health Organization , One Health. Key Facts, https://www.who.int/news‐room/fact‐sheets/detail/one‐health.

[156] S. Whitmee , A. Haines , C. Beyrer , et al., “Safeguarding Human Health in the Anthropocene Epoch: Report of The Rockefeller Foundation–Lancet Commission on planetary health,” Lancet 386 (2015): 1973–2028. 26188744 10.1016/S0140-6736(15)60901-1

[157] L. Persson , B. M. Carney Almroth , C. D. Collins , et al., “Outside the Safe Operating Space of the Planetary Boundary for Novel Entities,” Environmental Science & Technology 56 (2022): 1510–1521. 35038861 10.1021/acs.est.1c04158 PMC8811958

[158] A. Einstein , The Decade of Physics, 2nd ed., edited by D. Padovia (Hanser, n.d.), 55–56.

[159] K. R. Popper , The Logic of Scientific Discovery, 11th ed., edited by H. Keuth and M. Siebeck (Springer, 2005).

[160] European Union , Precautionary Principle, https://eur‐lex.europa.eu/EN/legal‐content/glossary/precautionary‐principle.html.

[161] D. Kriebel , J. Tickner , P. Epstein , et al., “The Precautionary Principle in Environmental Science,” Environmental Health Perspectives 109 (2011): 871–876. 10.1289/ehp.01109871 PMC1240435 11673114

[162] A. Kanai , “The ‘Simple Chemistry’ Programme for Sustainable Chemistry,” in Proceedings of the OECD Workshop on Sustainable Chemistry (OECD, 1998), 177.

[163] S. Böschen , D. Lenoir , and M. Scheringer , “Sustainabale Chemsitry: Starting Points and Prospects,” Naturwissenschaften 90 (2003): 93–102. 12649751 10.1007/s00114-002-0397-9

[164] K. Vranken and F. Nevens , Management Principles of Sustainable Industrial Chemistry: Theories, Concepts and Industrial Examples for Achieving Sustainable Chemical Products and Processes from a Non‐Technological Viewpoint, edited by G. L. Genserik , R. Sörensen , and K. Vrancken (Wiley VCH, 2013).

[165] P. Marion , B. Bernela , A. Piccirilli , et al., “Sustainable Chemistry: How to Produce Better and More from Less?,” Green Chemistry 19 (2017): 4973–4989.

[166] M. Richter , L. Vieira , and V. Sieber , “Sustainable Chemistry—An Interdisciplinary Matrix Approach,” ChemSusChem 14 (2020): 251–265, V. 32945148 10.1002/cssc.202001327

[167] A. R. Alcántara , P. Domínguez de María , J. A. Littlechild , M. Schürmann , R. A. Sheldon , and R. Wohlgemuth , “Biocatalysis as Key to Sustainable Industrial Chemistry,” ChemSusChem 15 (2021): e202102709. 10.1002/cssc.202102709 35238475

[168] H. Mutlu and L. Barner , “Getting the Terms Right: Green, Sustainable, or Circular Chemistry?,” Macromolecular Chemistry and Physics 223 (2022): 2200111.

[169] A. Cannon , S. Edwards , M. Jacobs , J. W. Moir , M. A. Roy , and J. A. Tickner , “An Actionable Definition and Criteria for “Sustainable Chemistry” Based on Literature Review and a Global Multisectoral Stakeholder Working Group,” RSC Sustainability 1 (2023): 2092–2106.

[170] The White House , The White House, Office of Science and Technology Policy (The White House, 2023), https://www.whitehouse.gov/ostp/newsupdates/2023/08/02/nstcsustainable‐chemistry‐report/.

[171] Stockholm Declaration , Read the Declaration (Stockholm Declaration, 2025), https://www.stockholm‐declaration.org/read‐the‐declaration.

[172] American Chemical Society , Green Chemistry ACS (American Chemical Society, 2024), accessed October 10, https://www.acs.org/greenchemistry.html.

[173] J. C. Slootweg , “Sustainable Chemistry: Green, Circular, and Safe‐by‐Design,” One Earth 7 (2024): 754–758.

[174] European Commission , Safe and Sustainable by Design (European Commission, 2026), accessed March 20, https://research‐and‐innovation.ec.europa.eu/research‐area/industrial‐research‐and‐innovation/chemicals‐and‐advanced‐materials/safe‐and‐sustainable‐design_en.

[175] L. G. Soeteman‐Hernández , C. Apel , B. Nowack , et al., “The Safe‐and‐Sustainable‐by‐Design Concept: Innovating Towards a More Sustainable Future,” Environmental Sustainability 7 (2024): 363–368.

[176] L. G. Soeteman‐Hernández , C. Apel , E. Strömberg , and K. Kümmerer , “Safe‐and‐Sustainable‐by‐Design (SSbD) as an Enabler—Positioning SSbD within Current Chemistry Frameworks,” Sustainable Chemistry and Pharmacy 50 (2026): 102321.

[177] IUPAC , Responsible Chemistry (IUPAC, 2025), accessed October 25, https://iupac.org/responsible‐chemistry/.

[178] A. King and M. Lenox , “Industry Self‐Regulation without Sanctions: The Chemical INDUSTRY'S Responsible Care Program,” Academy of Management Journal 43 (2000): 698–716, h.

[179] F. Gomollon‐Bel and J. García‐Martínez , “Connecting Chemical Worlds for a Sustainable Future,” Chemical Science 15 (2024): 5056–5060. 38577374 10.1039/d3sc06815c PMC10988580

[180] C. Blum , D. Bunke , M. Hungsberg , et al., “The Concept of Sustainable Chemistry: Key Drivers for the Transition towards Sustainable Development,” Sustainable Chemistry and Pharmacy 5 (2017): 94–104.

[181] K. Kümmerer and J. Clark , “Green and Sustainable Chemistry,” in Sustainability Science, edited by H. Heinrichs , P. Martens , G. Michelsen , and A. Wiek (Springer, 2016), 43–59.

[182] K. Kümmerer , “Sustainable Chemistry: A Future Guidiung Principle,” Angewandte Chemie International Edition 56 (2017): 16420–16421. 29111593 10.1002/anie.201709949

[183] K. Kümmerer , A. Amsel , D. Bartkowiak , A. Bazzanella , C. Blum , and C. Cinquemani , “Key Characteristics of Sustainable Chemistry. Towards a Common Understanding of Sustainable Chemistry,” (International Sustainable Chemistry Collaborative Centre, 2021), ISC3 Working Paper, https://isc3.org/cms/wp‐content/uploads/2022/06/ISC3_Sustainable_Chemistry_key_characteristics_20210113.pdf.

[184] OECD , “Economic Features of Chemical Leasing,” Series on Risk Management No. 37, Paris: Environment, Health and Safety, Environment Directorate (OECD, 2017).

[185] UNIDO , Chemicals Leasing. Function to Impact, a Performance‐Based Business Model for Sustainable Chemicals Management (UNIDO, 2020).

[186] M. Streek , “Chemical leasing—Efficient and Sustainable Hospital Hygiene,” DBU Abschlussbericht AZ 26035, 2025, https://opac.dbu.de/ab/DBU‐Abschlussbericht‐AZ‐26035.pdf.

[187] H. Zou , T. Wang , Z.‐L. Wang , and Z. Wang , “Continuing Large‐Scale Global Trade and Illegal Trade of Highly Hazardous Chemicals,” Nature Sustainability 6 (2023): 1394–1405.

[188] UNEP , The Bonn Declaration for a Planet Free of Harm From Chemicals and Waste (UNEP, 2023), https://www.unep.org/global‐framework‐chemicals/bonn‐declaration‐planet‐free‐harm‐chemicals‐and‐waste.

[189] K. Kümmerer , D. Dionysiou , O. Olsson , and D. Fatta‐Kassinos , “A Path to Clean Water,” Science 361 (2018): 222–224. 30026210 10.1126/science.aau2405

[190] J. de Boer and H. M. Stapleton , “Toward Fire Safety Without Chemical Risk,” Science 364 (2019): 231–232. 31000649 10.1126/science.aax2054

[191] J. M. Whalen , S. A. Matlin , T. A. Holme , J. J. Stewart , and P. G. A. Mahaffy , “A Systems Approach to Chemistry Is Required to Achieve Sustainable Transformation of Matter: The Case of Ammonia and Reactive Nitrogen,” ACS Sustainable Chemistry & Engineering 10 (2022): 12933–12947.

[192] S. A. Matlin , S. E. Cornell , K. Kümmerer , P. G. Mahaffy , and G. Mehta , “Inventing a Secure Future: Material Stewardship as Chemistry's Mission for Sustainability,” RSC Sustainability 3 (2025): 804–821.

[193] M. Elschami and K. Kümmerer , “Design of a Master of Science Sustainable Chemistry,” Sustainable Chemistry Pharmacy 17 (2020): 100270.

[194] G. A. Hurst and J. H. Clark , “Editorial Overview: Education in Green and Sustainable Chemistry and Green and Sustainable Pharmacy: An Integrated Approach,” Sustainable Chemistry Pharmacy 17 (2020): 100272.

[195] V. G. Zuin , I. Eilks , M. Elschami , and K. Kümmerer , “Education in Green Chemistry and in Sustainable Chemistry: Perspectives Towards Sustainability,” Green Chemistry 23 (2021): 1594–1608.

[196] M. Sivén , J. Teppo , O. Lapatto‐Reiniluoto , E. Teräsalmi , O. Salminen , and T. Sikanen , “Generation Green‐ A Holistic Approach to Implementation of Green Principles and Practices in Educational Programs in Pharmaceutical and Medical Sciences at the University of Helsinki,” Sustainable Chemistry Pharmacy 17 (2020): 100262.

[197] S. York and M. Orgill , “ChEMIST Table: A Tool for Designing or Modifying Instruction for a Systems Thinking Approach in Chemistry Education,” Journal of Chemical Education 97 (2020): 2114–2129.

[198] P. G. Mahaffy , S. A. Matlin , T. A. Holme , and J. MacKellar , “Systems Thinking for Education About the Molecular Basis of Sustainability,” Nature Sustainability 2 (2019): 362–370.

[199] R. Zidny and I. Eilks , “Integrating Perspectives From Indigenous Knowledge and Western Science in Secondary and Higher Chemistry Learning to Contribute to Sustainability Education,” Sustainable Chemistry and Pharmacy 17 (2020): 100229.

[200] N. Loste , E. Roldán , and B. Giner , “Is Green Chemistry a Feasible Tool for the Implementation of a Circular Economy?” Environmental Science and Pollution Research 27 (2020): 6215–6227. 31865584 10.1007/s11356-019-07177-5

[201] United Nations Environment Programme , Specialised Manual on Green and Sustainable Chemistry Education and Learning (UNEP, 2024), accessed October 25, https://www.unep.org/resources/report/specialized‐manual‐green‐and‐sustainable‐chemistry‐education‐and‐learning.

[202] C. Middlecamp , M. Kirchhoff , P. Mahaffy , and K. Kümmerer (eds.), Chemistry Education for a Sustainable Future, (Royal Society of Chemistry, 2025).

[203] A. Reihlen , D. Bunke , A. Gruhlke , R. Groß , and C. Blum , Guide on Sustainable Chemicals. A Decision Tool for Substance Manufacturers, Formulators and End Users of Chemicals, (Umweltbundesamt, 2016).

[204] Federal Environment Agency , SubSelect—Guide for the Selection of Sustainable Chemicals (Federal Environment Agency, 2020), www.uba.de/m47540en.

[205] C. Blum , B. Zeschmar‐Lahl , E. Heidbüchel , et al., “Metrics Are the Key: Development of Criteria and Indicators for Measuring Sustainability in International Chemicals Management,” RSC Sustainability 3 (2025): 4724–4745.

[206] G. Gnegel , Deutsche Apotheker Zeitung, PFAS als Wirkstoffe – auf der Suche nach Alternativen (Deutsche Apotheker Zeitung, 2015), https://www.deutsche‐apotheker‐zeitung.de/news/artikel/2023/04/04/pfas‐als‐wirkstoffe‐auf‐der‐suche‐nach‐alternativen.

